# Single-cell RNA-sequencing analysis identifies host long noncoding RNA MAMDC2-AS1 as a co-factor for HSV-1 nuclear transport

**DOI:** 10.7150/ijbs.42556

**Published:** 2020-03-05

**Authors:** Yiliang Wang, Lianzhou Huang, Yun Wang, Weisheng Luo, Feng Li, Ji Xiao, Shurong Qin, Zhaoyang Wang, Xiaowei Song, Yuan Wang, Fujun Jin, Yifei Wang

**Affiliations:** 1 College of Life science and Technology, Guangzhou Jinan Biomedicine Research and Development Center, Jinan University, Guangzhou 510632, PR China;; 2 College of Pharmacy, Jinan University, Guangzhou 510632, PR China;; 3 Department of Obstetrics and Gynecology, The First Affiliated Hospital of Jinan University, Guangzhou 510632, PR China;; 4 Integrated Chinese and Western Medicine Postdoctoral Research Station, Jinan University, Guangzhou 510632, PR China.

**Keywords:** single-cell RNA-sequencing, HSV-1, MAMDC2-AS1, tegument protein, nuclear transport, Hsp90α

## Abstract

Herpes simplex virus (HSV) type 1 (HSV-1) infection exhibited high heterogeneity at individual cells level, including the different gene expression patterns and varying amounts of progeny virus. However, the underlying mechanism of such variability remains obscure. The importance of host long noncoding RNAs (lncRNAs) in virus infection had been recognized, while the contribution of lncRNAs to the heterogeneous infection remains unknown. Herein, a prior single-cell RNA sequencing data using HSV-1 reporter strain expressing ICP4-YFP was re-analyzed to obtain the differentially expressed lncRNA between the successfully initiated viral gene expression (ICP4-YFP^+^) cells and the aborted infection cells (ICP4-YFP^-^). The ICP4-YFP^+^ population show a higher abundance of MAMDC2 antisense 1 (MAMDC2-AS1) lncRNA than ICP4-YFP^-^ population. MAMDC2-AS1 silencing reduces the expression of HSV-1 immediate early (IE) genes and limit HSV-1 infection in human host cells. Consistently, ectopic expression of MAMDC2-AS1 enhances HSV-1 IE genes transcription and facilitates the formation of HSV-1-induced plaques. Mechanically, both RNA-pull down and RNA immunoprecipitation assays show that MAMDC2-AS1 interacts with the RNA binding protein heat shock protein 90α (Hsp90α), a molecular chaperone involving in the nuclear import of HSV-1. The MAMDC2-AS1-Hsp90α interaction facilitates the nuclear transport of viral tegument protein VP16, the core factor initiating the expression of HSV-1 IE genes. The transcription factor YY1 mediates the induction of MAMDC2-AS1 upon HSV-1 infection. Our study elucidates the contribution of lncRNA to HSV-1 infection susceptibility in human cells and the role of Hsp90α RNA binding activity in HSV-1 infection.

## Introduction

Herpes simplex virus (HSV) type 1 (HSV-1) infection poses a major challenge to global health 1, 2. Traditional antiviral drugs against HSV-1 are limited to nucleoside analogs targeting viral factors; however, their long-term use has led to the frequent emergence of drug-resistant viruses 1. Novel antiviral strategy is urgently required to combat HSV-1 infection. As obligate parasites, the accomplishment of HSV-1 life cycle largely depends on host factors, suggesting that these host factors and their associated pathways may represent promising antiviral targets 1, 3-9. Such dependence on host factor partly contributed to the heterogeneous infection 10, while the mechanism mediating such great heterogeneity remains obscure. The advent of sing-cell RNA-sequencing has allowed researchers to study virus-host interactions 10, 11. HSV-1 exhibit a strictly regulated temporal cascade of gene expression that can be divided into three general stage: immediate-early (IE), early (E) and late (L) 5, 12, 13. IE genes (mainly include *α0*, *α4*, *α22*, *α27*, and *α47*) are the first set of HSV-1 genes to be transcribed and expressed in productively infected cells. Once HSV-1 genome was injected into nucleus, the tegument protein VP16 initiates the expression of HSV-1 IE genes as a core factor with the assistance of host cell factor 1 and octamer-binding transcription factor 1^12^. ICP4 is an ideal reporter gene for monitoring HSV-1 infection at earlier phase. In detail, ICP4 initiates the expression of E and L genes required for the accomplishment of HSV-1 life cycle in host cells 12. A sing-cell RNA-sequencing has been performed towards the ICP4-positive and ICP4-negative cell population sorting from primary human fibroblasts (HDFn) infecting the reporter virus YFP-HSV-1 that was genetically modified to express ICP4-YFP 10, 11. Despite these studies revealed the transcriptional heterogeneity of coding factors during HSV-1 infection 11, the contribution of long non-coding RNAs (lncRNAs) to the inter-individual difference upon HSV-1 infection remains unknown. Indeed, lncRNAs play crucial roles in the process of HSV-l infection 14, 15. HSV-1 infection also alters the expression profile from the host cell genome, especially the expression of antisense transcripts 16-19. In general, lncRNAs function in viral infection primarily through two manners, one being dependent of the antiviral response by the host and the other being antiviral response-independent, such as cellular metabolism, maintaining the stability of viral factors and facilitating the expression of viral genes 16, 20-24. For example, NEAT1 enhances the expression of antiviral factors such as interleukin (IL)-8; this is mediated by the relocation of SFPQ from the promoter region to paraspeckles and results in the regulation of IL-8 expression in response to virus infection 25. lncRNAs can also be utilized by virus to facilitate the expression of viral genes and maintain the stability of viral factors 26-29. For instance, HSV-1 infection drastically increases the level of NEAT1 enhancing viral IE genes transcription and viral replication by the binding of several paraspeckle components 27. NEAT1 knockdown also enhances HIV-1 production by facilitating the nucleus-to-cytoplasm export of Rev-dependent instability element-containing HIV-1 mRNA 28, 29. However, further investigation is required to explore the contribution of lncRNAs in the inter-individual difference in response to HSV-1 infection.

Herein, based on the sing-cell RNA-sequencings data, MAMDC2 antisense 1 (MAMDC2-AS1) lncRNA was found to be a factor facilitating the initiation of HSV-1 IE genes thorough interacting Hsp90α. Hsp90α is a newly identified RNA binding protein with conserved RNA-binding regions across homologous while the function of its RNA binding activity in virus infection remains unknown. Our study shed novel insight into the role of Hsp90α in HSV-1 infection and uncovers the contribution of lncRNA to the varying response upon HSV-1 infection.

## Results

### MAMDC2-AS1 is a lncRNA associated with HSV-1 IE genes expression

**Figure 1:**
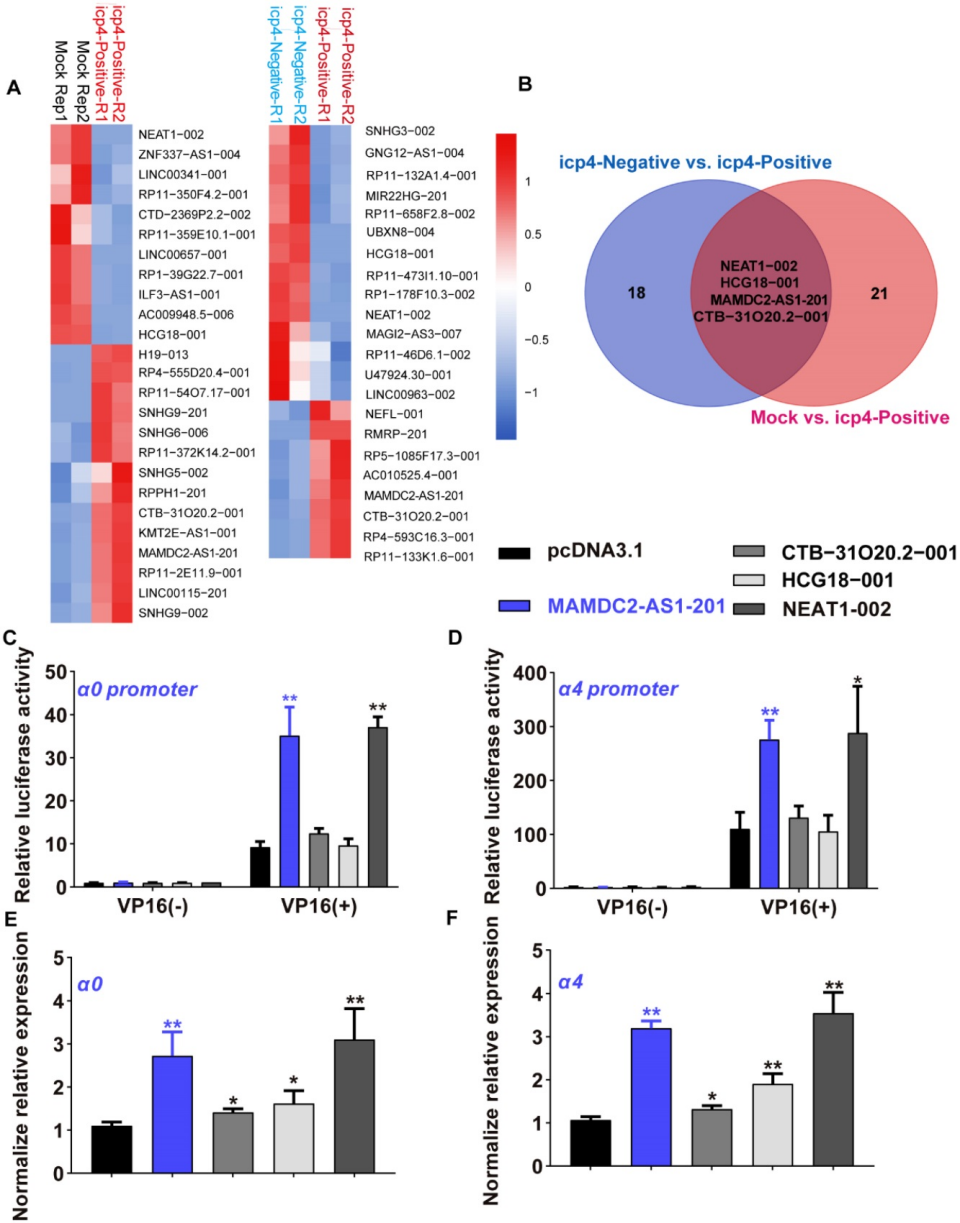
** Single-cell RNA-sequencing identified MAMDC2-AS1 as a lncRNA associated with HSV-1 IE genes expression. (A)** Heatmap of relative expressions of differentially expressed lncRNAs (DELs) in the comparison of indicated groups. HSV-1 infected HDFs were sorted into ICP4-negative and ICP4-positive using FACS based on gene expression pattern of YFP. The population expressing top 30% of YFP expression was defined ICP4-positive cells. The population expressed similar level of YFP expression with mock-infected cells were defined as ICP4-negative cells. **(B)** Venn diagram analysis (https://bioinfogp.cnb.csic.es/tools/venny/index.html) for the result of (A) to obtain the overlapped DELs in both comparisons, including ICP4-positive vs. Mock and ICP4-negative vs. ICP4-positive; **(C)** Effect of indicated lncRNA on *α0* promoter activity. 293T cells were co-transfected expressed plasmids of corresponding lncRNA and reporter plasmids indicating the promoter activity of HSV-1 *α0* gene for 24h. To activate *α0* promoter, the plasmids expressing VP16 were also co-transfected in all our dual luciferase assay given that VP16 is the core factor of HSV-1 IE genes transcription. Cells were harvested and the cell lysates were subjected to test luciferase activity as described in *Materials and Methods*. Bar graph represents the result of DLRs from 3 independent experiments expressed as mean value ± standard deviation (SD); **(D)** Effect of indicated lncRNA on α4 promoter activity. 293T cells were co-transfected plasmids expressing indicated lncRNA and reporter plasmids indicating the promoter activity of HSV-1 *α4* gene for 24h as described in Materials and Methods. Cells were harvested and the cell lysates were subjected to detect luciferase activity. Bar graph represents the result of DLRs from 3 independent experiments expressed as mean ± S.D; **(E)** Effects of indicated lncRNA on the mRNA expression of α0 in the context of HSV-1 infection; 293T cells were transfected with plasmids(1.5μg) expressing indicated lncRNA for 24h and then infected with HSV-1 (MOI 3). Total RNA was extracted at 2 h.p.i then subjected to analyze the level of α0 using qRT-PCR. **(F)** Effects of indicated lncRNA on the mRNA expression of α4 in the context of HSV-1 infection; 293T cells were transfected with plasmids(1.5μg) expressing indicated lncRNA for 24h and then infected with HSV-1 (MOI 3). Total RNA was extracted at 2 h.p.i then subjected to analyze the level of α4 using qRT-PCR.

To determine the lncRNA associated with HSV-1 infection susceptibility, the data of single-cell RNA-sequencing were re-analyzed and obtained 22 annotated differentially expressed lncRNAs (DELs) between mock cells group and ICP4-positve cells (**Figure 1A**). There are also 25 DELs with annotation between ICP4-positive and ICP4-negative cells group (**Figure 1A**). As present by the Venn diagram, only four DELs overlapped in both comparisons (**Figure 1B**), implying the potential functions of these four DELs in initiating the expression of HSV-1 IE genes, especially *α4* gene 5, 12. To determine the effect of these DELs on HSV-1 IE genes initiation, we first constructed the plasmids of expressing these four lncRNAs then tested their effect on HSV-1 *α0* and* α4* promoter activity with dual luciferase assay as established by our prior study 5. Of note, only the overexpression of lncRNA NEAT1-002 and MAMDC2-AS1-201 significantly enhanced the promoter activity of HSV-1 *α0* and *α4* in human 293T cells (**Figure 1C and D**). In contrast, both CTB-31O20.2-001 and HCG18-001 were failed to affect the promoter activity of *α0* and *α4* (**Figure 1C and D**). Consistently, NEAT1-002 and MAMDC2-AS1-201 also facilitated the mRNA expression of HSV-1 IE genes, including *α0* and *α4* (**Figure 1E and F**). Both CTB-31O20.2-001 and HCG18-001 lncRNA also exhibited a minor effect on the mRNA expression of HSV-1 *α0* and *α4* gene (**Figure 1E and F**). Collectively, MAMDC2-AS1-201 and NEAT1-002 are host lncRNAs associated with HSV-1 IE genes expression. However, given prior study has revealed the role of lncRNA NEAT1 in facilitating the expression of HSV-1 IE genes 27, we focused on studying MAMDC2-AS1.

### HSV-1 infection increases the expression of MAMDC2-AS1

**Figure 2:**
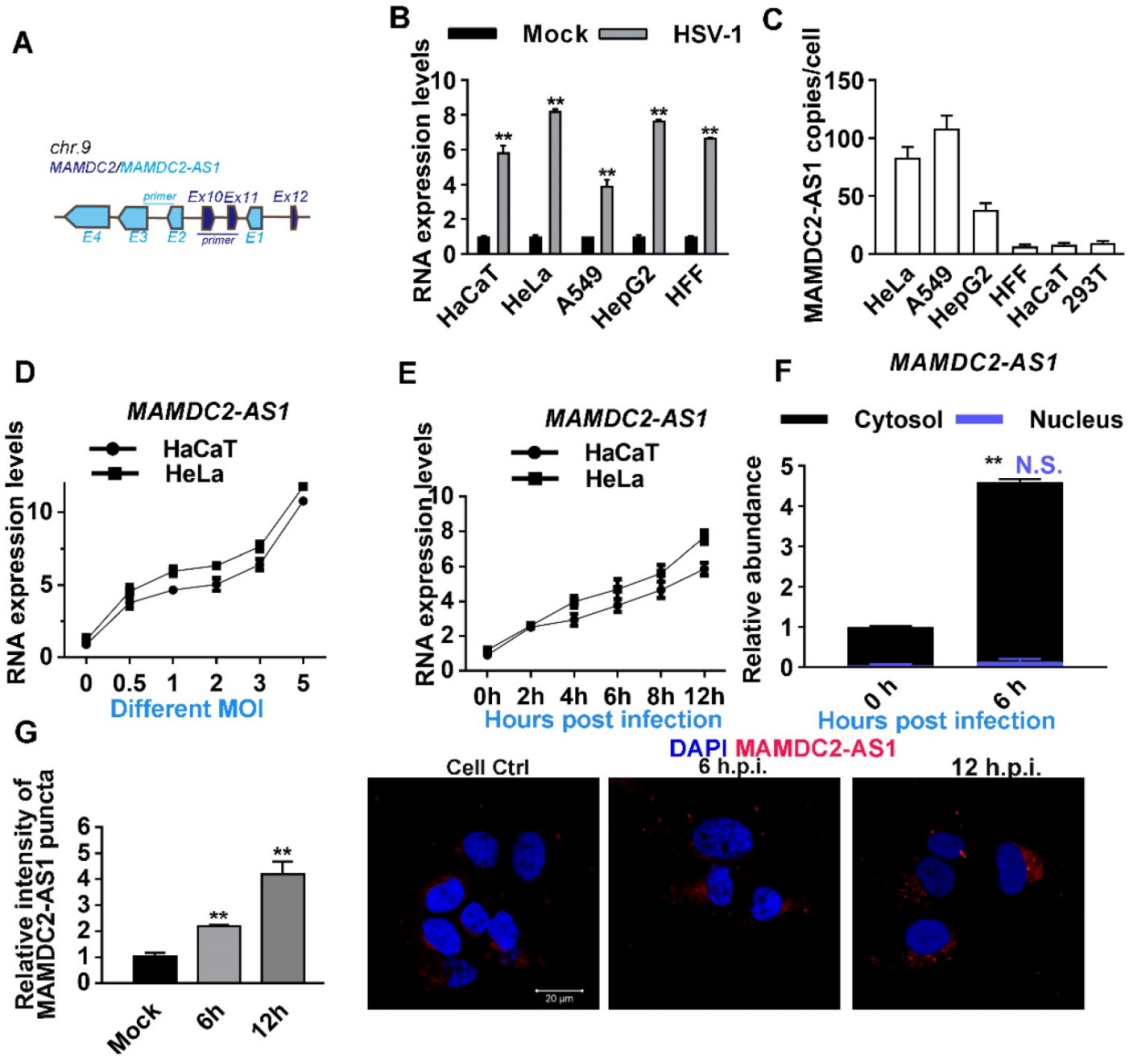
**HSV-1 infection increases the expression of MAMDC2-AS1. (A)** Schematic showing the genes *MAMDC2-AS1* and *MAMDC2* in distinct colors, as well as the primers targeting specific sites to differentiate between the genes. **(B)** MAMDC2-AS1 was significantly upregulated upon HSV-1 infection in different cells. After plating, the indicated cells were infected with HSV-1 (MOI 3), and total RNA was harvested at 12 h after HSV-1 infection to measure MAMDC2-AS1 expression by using qRT-PCR. **(C)** Absolute qRT-PCR analysis of MAMDC2-AS1 in indicated cell lines. The decimal serial dilutions of pcDNA3.1-MAMDC2-AS1 plasmids were used to describe standard curves. Total RNA was isolated from equal amount of indicated cells then subjected to analyze with absolute qRT-PCR to determine the copy number of MAMDC2-AS1 per cell. **(D)** HSV-1 infection facilitated the expression of MAMDC2-AS1 in a virus dose-dependent manner. The indicated cells were infected with HSV-1 at the specified MOIs for 12 h, and then, total RNA was extracted for analyzing the MAMDC2-AS1 level by qRT-PCR. **(E)** MAMDC2-AS1 expression following HSV-1 infection was increased in an infection time-dependent manner. HaCaT and HeLa cells were infected with HSV-1 (MOI 3) for the indicated periods, and then, total RNA was isolated for qRT-PCR analysis of MAMDC2-AS1 level. **(F)** Relative qRT-PCR analysis of the level of MAMDC2-AS1 in nuclear and cytoplasmic fractions from HeLa cells with HSV-1 infection for 6 h or without. Detailed information regarding such experiment can be obtained from Materials and Methods. **(G)** RNA FISH imaging of MAMDC2-AS1 after HSV-1 infection for the indicated durations. HeLa cells were fixed at the indicated durations after HSV-1 infection and then incubated with the MAMDC2-AS1 probe (red); nuclei were labeled with DAPI (blue). Fluorescence images were obtained using a confocal microscope. The average intensity of MAMDC2-AS1 puncta was analyzed with Zen software after acquiring images for three fields of view per dish. All quantitative results were obtained from three independent experiments with three technical replicates per experiments and one representative result was presented as mean ± SD.

The function of MAMDC2-AS1 in the initiation of HSV-1 *α0* and *α4* transcription inspired us to determine the relationship between HSV-1 infection and MAMDC2-AS1. *MAMDC2-AS1* partially overlaps with the coding gene MAM domain-containing 2 (*MAMDC2*) within introns 19 and 20. We used primers that selectively targeted corresponding sites in MAMDC2-AS1, as indicated, to ensure specific amplification in the detection of indicated genes (**Figure 2A**). Indeed, prior RNA-seq data also revealed that MAMDC2-AS1 is markedly upregulated upon HSV-1 infection in human foreskin fibroblasts cells 16, 19. We first determined the expression of MAMDC2-AS1 during the process of HSV-1 infection in multiple human cell lines, including HaCaT, HeLa, A549, and HepG2 cells. Consistent with RNA-seq results, the analysis of qRT-PCR showed that MAMDC2-AS1 significantly upregulated upon HSV-1 infection in different human cell lines (**Figure 2B**). Absolute quantification results revealed that both A549 and HeLa cells harbored with most abundant MAMDC2-AS1 among the cell lines we tested (**Figure 2C**). Moreover, the upregulation of MAMDC2-AS1 upon HSV-1 infection showed a doses-dependent manner (**Figure 2D**). Also, HSV-1 infection stimulated the expression of MAMDC2-AS1 in an infection times-dependent manner in both HaCaT and HeLa cells (**Figure 2E**). The qRT-PCR analysis of subcellular fraction showed that MAMDC2-AS1 was mainly located in cytosol and HSV-1 infection mainly induced the increment of MAMDC2-AS1 in cytosol but not in nucleus (**Figure 2F**). Consistently, the results of RNA fluorescence *in situ* hybridization (FISH) assays further demonstrated that HSV-1 infection increased the puncta of MAMDC2-AS1 in different times post infection (**Figure 2G**). Collectively, HSV-1 infection-triggered upregulation of MAMDC2-AS1 is a general response upon HSV-1 infection among different cell lines.

### MAMDC2-AS1 facilitates HSV-1 infection

**Figure 3:**
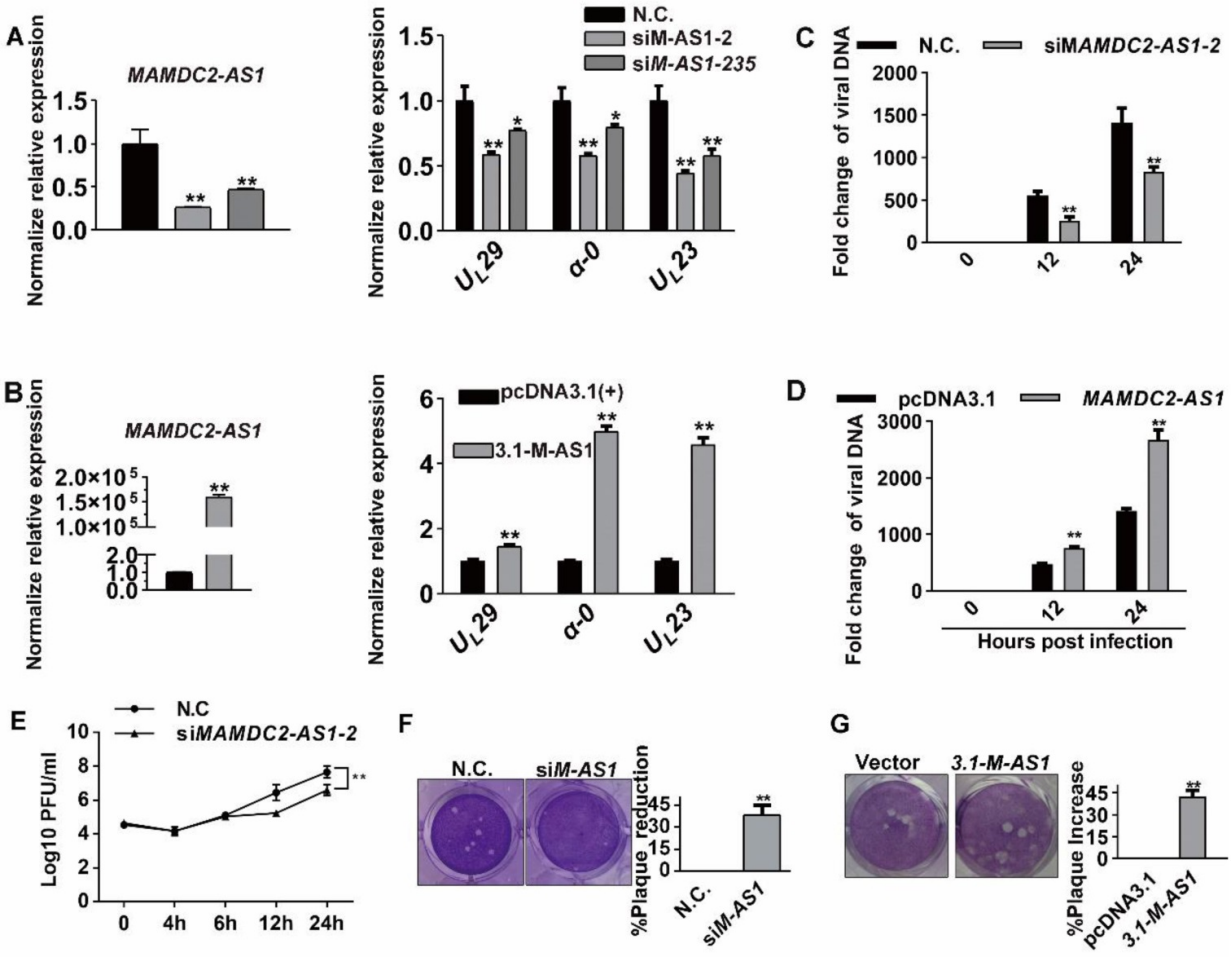
** MAMDC2-AS1 facilitates HSV-1 infection. (A)** MAMDC2-AS1 knockdown reduced the expression of HSV-1 genes. HeLa cells transfected with MAMDC2-AS1-targeting siRNA (siMAMDC2-AS1, 100 nM) or with a negative-control (N.C.) siRNA for 24 h and then infected with HSV-1 (MOI 3) for another 10 h. Total RNA was extracted to analyze the mRNA level of indicated genes by qRT-PCR. **(B)** MAMDC2-AS1 overexpression increased the level of HSV-1 genes. HeLa cells transfected with the plasmid pcDNA3.1-MAMDC2-AS1 (1.5 μg) or empty vector pcDNA3.1 plasmid (1.5 μg) for 24 h were infected with HSV-1 (MOI 3) for 10 h. Total RNA was extracted for qRT-PCR analysis to determine the relative mRNA expression of indicated genes. **(C)** MAMDC2-AS1 knockdown reduced the replication of HSV-1 genome. HeLa cells transfected with siM-AS1-2 or with a N.C. siRNA (100 nM), were infected with HSV-1 (MOI 3) for indicated hours. Viral DNA was extracted then the relative level of HSV-1 DNA was quantified using qRT-PCR with the primers targeting U_L_47. **(D)** MAMDC2-AS1 overexpression facilitated the replication of HSV-1 genome. HeLa cells transfected with the plasmid pcDNA3.1-MAMDC2-AS1 (1.5 μg) or empty vector pcDNA3.1 plasmid (1.5 μg) for 24 h were infected with HSV-1 (MOI 3) for indicated hours. Viral DNA was extracted after freeze-thawing thrice, and the relative level of viral DNA was determined using qRT-PCR with the primers targeting U_L_47. **(E)** Viral growth curve assay in MAMDC2-AS1-knockdown and control cells. HeLa cells transfected with siM-AS1-2 or with a N.C. siRNA (100 nM) were infected with HSV-1 (MOI 1) for indicated hours. One well of each culture was collected (along with medium) at indicated time points and then stored at -80°C. After freeze-thawing thrice, viral titer was determined according to the method of “Viral plaque assay”. **(F)** Plaque-formation assay results showing that MAMDC2-AS1 knockdown limited HSV-1 infection. HeLa cells transfected with siM-AS1-235 or with a N.C. siRNA (100 nM) for 24 h were infected with HSV-1 (MOI 0.01) for 2 h, and then the cells were overlaid with medium containing 1% serum, and after 72 h, the reduction in plaque formation was examined (left) and quantified (corresponding histograms, right). **(G)** Plaque-formation assay results showing that MAMDC2-AS1 overexpression facilitated HSV-1 infection. HeLa cells transfected with pcDNA3.1-MAMDC2-AS1 or empty vector pcDNA3.1 plasmid (500 ng) for 24 h were infected with HSV-1 (MOI 0.01) for 2 h; and after 72 h, the increment in plaque formation was examined (left) and quantified histograms(right). All quantitative results were obtained from three independent experiments with three technical replicates per experiments and one typical result was presented as means and SD.

To clarify the function of MAMDC2-AS1 in HSV-1 infection, two siRNAs that most efficiently knocked down MAMDC2-AS1 expression (nos. 2 and 235) were utilized. Notably, virus genes, including *α-0*,* U_L_23* and *U_L_29*, were downregulated in the context of MAMDC2-AS1 knockdown (**Figure 3A**). The two distinct siRNAs yielded similar results, making it highly unlikely that the observed effect was produced by a nonspecific siRNA-dependent mechanism. Inhibition effect of MAMDC2-AS1 knockdown on viral genes expression was also found in HepG2 cells (**Figure S1**). Conversely, MAMDC2-AS1 overexpression led to a substantially increasement of viral gene expression upon HSV-1 infection (**Figure 3B**). Next, we analyzed the amount of viral DNA at continuous hours post infection in the context of MAMDC2-AS1 knockdown or overexpression. We found that MAMDC2-AS1 knockdown reduced the amount of viral DNA at different times post infection in host cells (**Figure 3C**), while MAMDC2-AS1 overexpression led to an increment of viral DNA amount (**Figure 3D**). The viral growth curves also indicated that knockdown of MAMDC2-AS1 delayed HSV-1 growth rate (**Figure 3E**). Moreover, the amount of HSV-1 infection-mediated plaque formation units is significantly reduced by MAMDC2-AS1 knockdown (**Figure 3F**). Consistently, MAMDC2-AS1 overexpression facilitated the formation of plaques induced by HSV-1 infection (**Figure 3G**). Furthermore, infection of MAMDC2-AS1 knockdown cells with enhanced green fluorescent protein (EGFP)-HSV-1, another HSV-1 strain that harbors *EGFP-*tagged *U_S_11*, yielded EGFP fluorescence at a lower intensity than that of infected control cells (**Figure S2**). Taken together, MAMDC2-AS1 serve a beneficial function for HSV-1 infection.

### MAMDC2-AS1 does not function through regulating antiviral response factors or affect HSV-1 attachment and entry into cells

**Figure 4:**
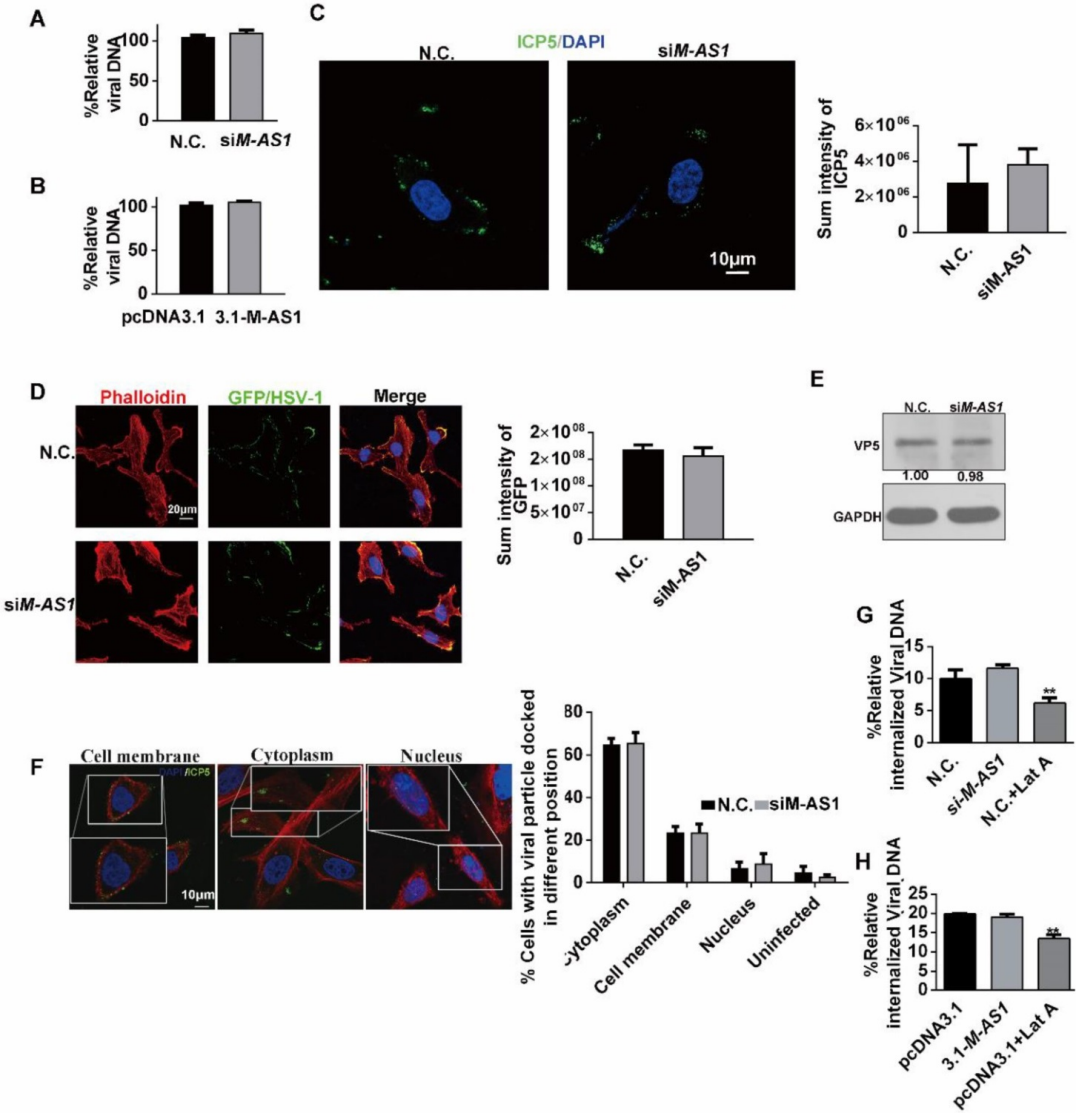
** MAMDC2-AS1 does not function through regulating antiviral response factors or affect HSV-1 attachment and entry into cells. (A)** MAMDC2-AS1 knockdown did not affect viral attachment. HeLa cells were incubated with HSV-1 (MOI 3) at 4°C for 2 h after transfection with siM-AS1-235 or N.C. siRNA (100 nM); samples were collected for extracting viral DNA to determine HSV-1 binding amount according to the method of “Detection of HSV-1 binding and internalization”. **(B)** MAMDC2-AS1 overexpression did not affect virus binding to cells. HeLa cells were transfected with pcDNA3.1-MAMDC2-AS1 or empty vector pcDNA3.1 plasmid (1.5 µg) for 24 h and then infected with HSV-1 (MOI 3) at 4°C for 2 h; samples were collected for extracting viral DNA to detect HSV-1 binding amount according to the method of “Detection of HSV-1 binding and internalization”. **(C)** Confocal imaging of viral attachment (upper panel) and the corresponding quantitative results (histogram, lower panel). HeLa cells were transfected with siM-AS1-235 or N.C. siRNA (100 nM) for 24 h and then infected with HSV-1 (MOI 50) at 4°C for 2 h. The samples were fixed, after which ICP5 and nuclei were labeled with the ICP5-specific antibody (green) and DAPI (blue), respectively. The cells were examined using a confocal microscope, and the average fluorescence intensity of ICP5 labeling was quantified using Zen software. **(D)** Confocal imaging results (upper) showing similar EGFP fluorescence intensity (lower) in control cells and MAMDC2-AS1 knockdown cells infected with EGFP-HSV-1. HeLa cells were transfected with MAMDC2-AS1*-235* or N.C. siRNA (100 nM) for 24 h and then infected with EGFP-HSV-1 (MOI 50) at 4°C for 2 h, after which the cells were fixed and nuclei labeled with DAPI. F-actin were labelled with phalloidin to indicated the cellular contour. These samples were observed by confocal microscopy, and the average fluorescence intensity of EGFP was calculated using Zen software. **(E)** Corresponding ICP5 protein levels of the protein samples (80 μg) that were the same as those in (C), determined by western blotting. **(F)** Confocal imaging of viral particles located at the indicated sites (right) and the percentage of viral particle at indicated sites (histogram, left). HeLa cells were transfected with si*M-AS1*-235 or N.C. siRNA (100 nM) for 24 h and then infected with HSV-1 (MOI 50) at 37°C for 10 min, after which the samples were fixed and ICP5 and nuclei labeled with the ICP5-specific antibody (green) and DAPI (blue), respectively. Confocal microscopy was performed, and at least 50 cells were counted in each experiment and quantified into the proportion of different sites (histogram). **(G)** MAMDC2-AS1 knockdown did not significantly affect HSV-1 internalization. HeLa cells were transfected with si*M-AS1*-235 or N.C. siRNA (100 nM) for 24 h and then infected with HSV-1 (MOI 50) for 30 min at 37°C. As a positive control, cells were infected with HSV-1 in the presence of Lat-A (0.25 μM), a cytoskeleton inhibitor known to inhibit HSV-1 internalization. Subsequently, viral DNA was extracted to detect the amount of HSV-1 internalization according to the method of “Detection of HSV-1 binding and internalization”. **(H)** MAMDC2-AS1 overexpression did not markedly affect HSV-1 internalization. HeLa cells were transfected with pcDNA3.1-MAMDC2-AS1 or empty vector pcDNA3.1 plasmid (1.5 μg) for 24 h and then infected with HSV-1 (MOI 50) for 30 min at 37°C. Finally, viral DNA was extracted to determine the amount of HSV-1 internalization according to the method of “Detection of HSV-1 binding and internalization”. All quantitative results were obtained from three independent experiments with three technical replicates per experiments and one typical result was presented as means and S.D.

Previous studies have suggested that lncRNA can modulate the expression of interferon-stimulated genes (ISGs) affecting HSV-1 infection 23-25. Thus, we first tested the possibility of regulating the expression of ISGs by MAMDC2-AS1. However, we found that MAMDC2-AS1 knockdown, which impaired HSV-1 replication (above), also reduced the levels of antiviral factors *IFITM1*, *IFITM2*, *ISG15*, *ISG56*, and *TNFα* (**Figure S3A**). In contrast, MAMDC2-AS1 overexpression increased the expression of these genes, including *IFITM1*, *IFITM2*, *ISG56*, and *TNFα* (**Figure S3B**). However, MAMDC2-AS1 knockdown did not exhibit such effects on the level of ISGs that stimulated by Toll-like receptor 4 agonist LPS (**Figure S4**). Collectively, the inhibition effect on the expression of these ISGs may not be the cause of limiting HSV-1 infection by MAMDC2-AS1 knockdown. Next, to determine whether MAMDC2-AS1 functions through autophagy, a pathway involved in viral clearance 30, 31, we tested the autophagosome marker LC3B by confocal microscopy. The results showed that MAMDC2-AS1 knockdown did not affect the accumulation of LC3B-II puncta** (Figure S5).** Collectively, MAMDC2-AS1 potentially functions in HSV-1 infection in a manner independent of the host innate immune response and autophagy initiation. We next tested the direct effect of MAMDC2-AS1 on the progression of HSV-1 life cycle, which consists of several crucial phases, including cell attachment, capsid entry into cytoplasm, regiment protein transport into nucleus, and viral gene transcription. We first investigated the amount of HSV-1 bound to cells by quantifying the viral genome, a common method that used for determining the amount of viral attachment during early infection 32-34. The results indicated that the amount of viral attachment was comparable to control groups after either MAMDC2-AS1 knockdown (**Figure 4A**) or MAMDC2-AS1 overexpression (**Figure 4B**). Moreover, the fluorescence intensity of ICP5 labeling in the context of MAMDC2-AS1 knockdown did not exhibit a significant difference from that in control cells at the phase of attachment (**Figure 4C**). Infection with EGFP-HSV-1 also generated a similar EGFP intensity as that in control group (**Figure 4D**). Collectively, MAMDC2-AS1 did not affect viral attachment. Therefore, we next determined whether MAMDC2- AS1 affects the subsequent phase of HSV-1 life cycle, i.e. virus internalization. We subsequently analyzed the proportion of viral particles located in the cytoplasm by examining ICP5 localization at 30 min post infection 35, 36. The results showed that viral capsid proteins were mainly localized in the cytoplasm, and such distribution was not affected by MAMDC2-AS1 (**Figure 4E**). Moreover, MAMDC2- AS1 knockdown did not affect the level of ICP5 as demonstrated by the results of western blotting (**Figure 4F**). Furthermore, the internalized viral DNA amount at the same time point did not differ significantly between MAMDC2-AS1-knockdown and controls cells (**Figure 4G**). Accordingly, MAMDC2-AS1 overexpression indicated identical amounts of virus internalization as compared to control cells (**Figure 4H**). Collectively, MAMDC2-AS1 did not affect the attachment and internalization of HSV-1 to host cells.

### MAMDC2-AS1 facilitates the nuclear translocation of HSV-1 tegument protein VP16

**Figure 5:**
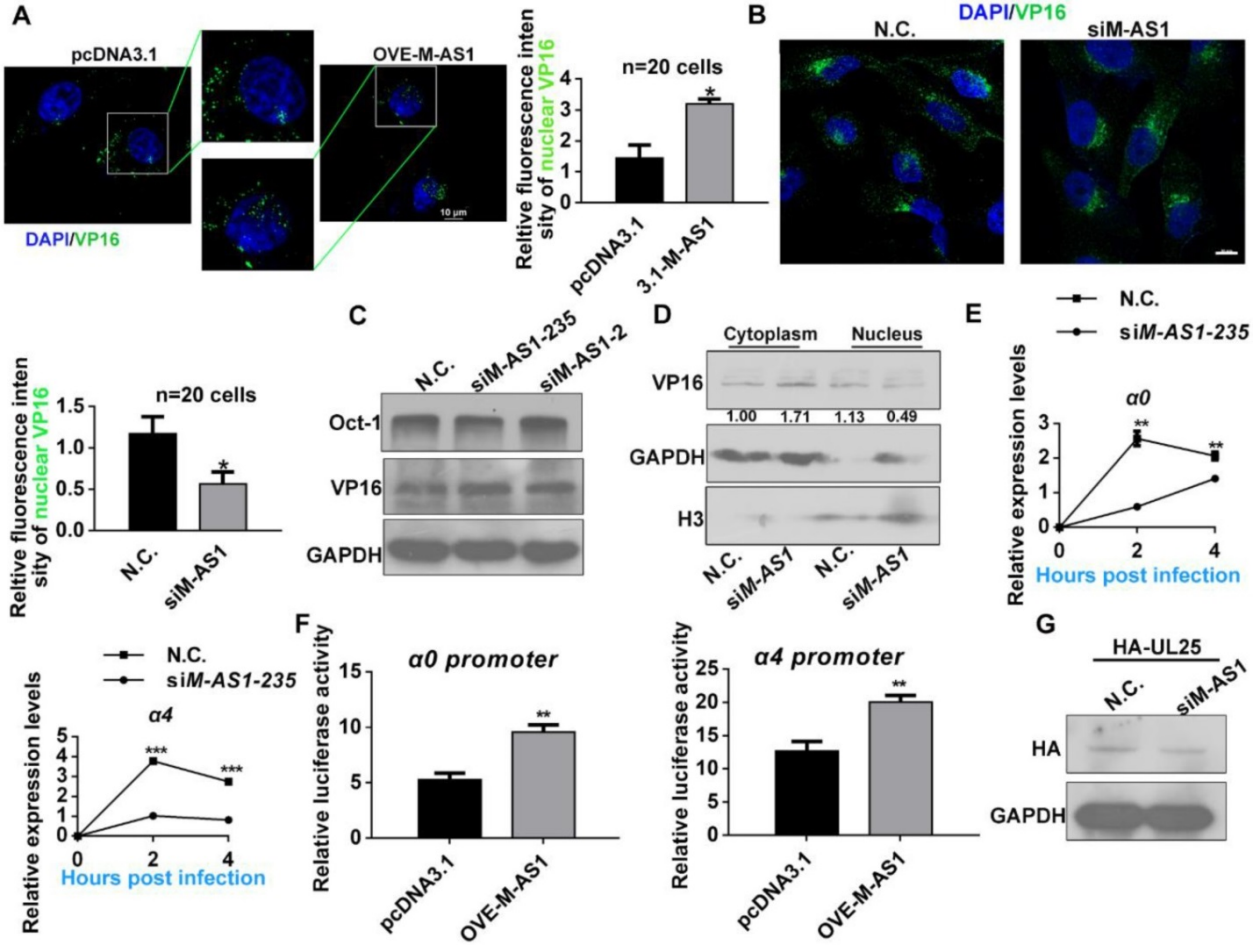
** MAMDC2-AS1 facilitates the nuclear translocation of HSV-1 tegument protein VP16. (A)** Nuclear docking of VP16 in MAMDC2-AS1 overexpression cells. HeLa cells were transfected pcDNA3.1-MAMDC2-AS1 or empty vector pcDNA3.1 plasmid (1.5 µg) for 24 h and then pretreated with cycloheximide (100 μg/mL) for 2 h follow by infected with HSV-1 (MOI 100) for 2 h, after which the cells were fixed and VP16 and nuclei were labeled with VP16-specific antibody (green) and DAPI (blue), respectively. The corresponding quantitative results of VP16 fluorescence intensity in nuclear were prepared as shown in the histogram (n = 20 cells); **(B)** Nuclear docking of VP16 in MAMDC2-AS1 knockdown cells. HeLa cells were transfected with siM-AS1-235 or N.C. siRNA (100 nM) for 24 h and then infected with HSV-1 (MOI 100) for 2 h, after which the cells were fixed and VP16 and nuclei labeled with the VP16-specific antibody (green) and DAPI (blue), respectively. The corresponding quantitative results of VP16 puncta in nuclear were prepared as shown in the histogram (n = 20 cells). **(C)** MAMDC2-AS1 knockdown did not alter Oct-1 and VP16 levels. HeLa cells were transfected with siM-AS1 or N.C. siRNA (100 nM) targeting different sites for 24 h and then infected with HSV-1 (MOI 100) for 2 h, after which total-protein extracts were analyzed for the levels of the indicated factors. **(D)** VP16 nuclear levels diminished in the context of MAMDC2-AS1 knockdown. HeLa cells were infected with HSV-1 (MOI 100) for 2 h after transfection with siM-AS1-235 or N.C. siRNA (100 nM). The cells were fractionated into cytoplasmic and nuclear fractions, which were loaded at a 1:2 ratio for SDS-PAGE, followed by western blotting analysis of the levels of the indicated molecules. GAPDH and H3 were used as loading controls for cytoplasmic and nuclear fractions, respectively. **(E)** Expression of the HSV-1 IE genes, including *α0* and *α4*, in MAMDC2-AS1 knockdown cells at the indicated infection times. HeLa cells were infected with HSV-1 (MOI 3) for the indicated durations after transfection with siM-AS1 or N.C. siRNA (100 nM), and then, total RNA was extracted for qRT-PCR analysis of the levels of *α0* and *α4*. **(F)** Activity of HSV-1 *α0* and *α4* promoter in the context of MAMDC2-AS1 overexpression. HeLa cells were co-transfected with pcDNA3.1-MAMDC2-AS1 or empty vector pcDNA3.1 plasmid (1.5 μg), the reporter plasmid pGL-α0-promoter (500 ng) or pGL-α4-promoter (500 ng), and the pcDNA-VP16 plasmid (500 ng) for 24 h; subsequently, sample lysates were prepared, and luciferase activity was analyzed. **(G)** MAMDC2-AS1 knockdown did not affect the level of UL25 expressed from plasmids. HeLa cells were co-transfected with siM-AS1-235 or N.C. siRNA (100 nM) and HA-UL25 plasmid (3 μg) for 48 h, after which total-protein extracts were analyzed for the level of HA-UL25. All quantitative results were obtained from three independent experiments with three technical replicates per experiments and one typical result was presented as means and S.D.

After viral genome was transported by capsid proteins and then injected into the nucleus, HSV-1 tegument protein VP16 enters into nucleus then initiates the transcription of HSV-1 α genes with the assistance of host factor Oct-1 12, 13. Then, we investigated the effect of MAMDC2-AS1 on the nuclear import of HSV-1 tegument protein VP16 and the injection viral DNA to nucleus. We first evaluated a continued times post infection to determine the appropriate time at which most of VP16 could be detected in the nucleus by confocal microscopy. The results revealed that most viral particles entered the nucleus 2 h after HSV-1 infection at a MOI of 100 (data not show). Thus, we analyzed the effect of MAMDC2-AS1 on the nuclear transport of HSV-1 tegument protein at 2 h post infection. The protein translational inhibitor cycloheximide (CHX) was utilized to remove the interruption of newly synthesized VP16. The image of confocal microscopy demonstrated that MAMDC2-AS1 overexpression facilitated the nuclear translocation of VP16 (**Figure 5A**). Consistently, MAMDC2-AS1 knockdown limited the nuclear transport of VP16 (**Figure 5B**), but did not affect the protein level of VP16 and Oct-1, both of which are crucial factors participating in the transcription of HSV-1 α genes (**Figure 5C**). Furthermore, we detected the level of VP16 in the cytoplasm and nucleus followed by isolating subcellular fraction extraction, and found that MAMDC2-AS1 knockdown led to an accumulation of VP16 in the cytoplasm and reduced the level of VP16 in the nucleus (**Figure 5D**). We also observed a significant reduction in the level of transcripts expressed from α genes, including α0 and *α4*, at different times post infection in the context of MAMDC2-AS1 knockdown (**Figure 5E**).Given VP16 initiates the expression of HSV-1 α genes via binding the promoter of *α0* and *α4* genes, dual-luciferase assays were performed to determine the effect of MAMDC2-AS1 on their promoter activity. The results of dual luciferase assay indicated that MAMDC2-AS1 overexpression enhances the promoter activity of *α0* and *α4* genes in HeLa cells (**Figure 5F**). However, the downregulation of MAMDC2-AS1 did not affect the level of U_L_25 (**Figure 5G**), a factor involving in viral DNA release 37. Collectively, MAMDC2-AS1 regulates the nuclear transport of viral tegument protein VP16, thereby modulating the expression of HSV-1 α genes.

### MAMDC2-AS1 interacts with Hsp90α to facilitate the nuclear import of HSV-1 tegument protein

**Figure 6:**
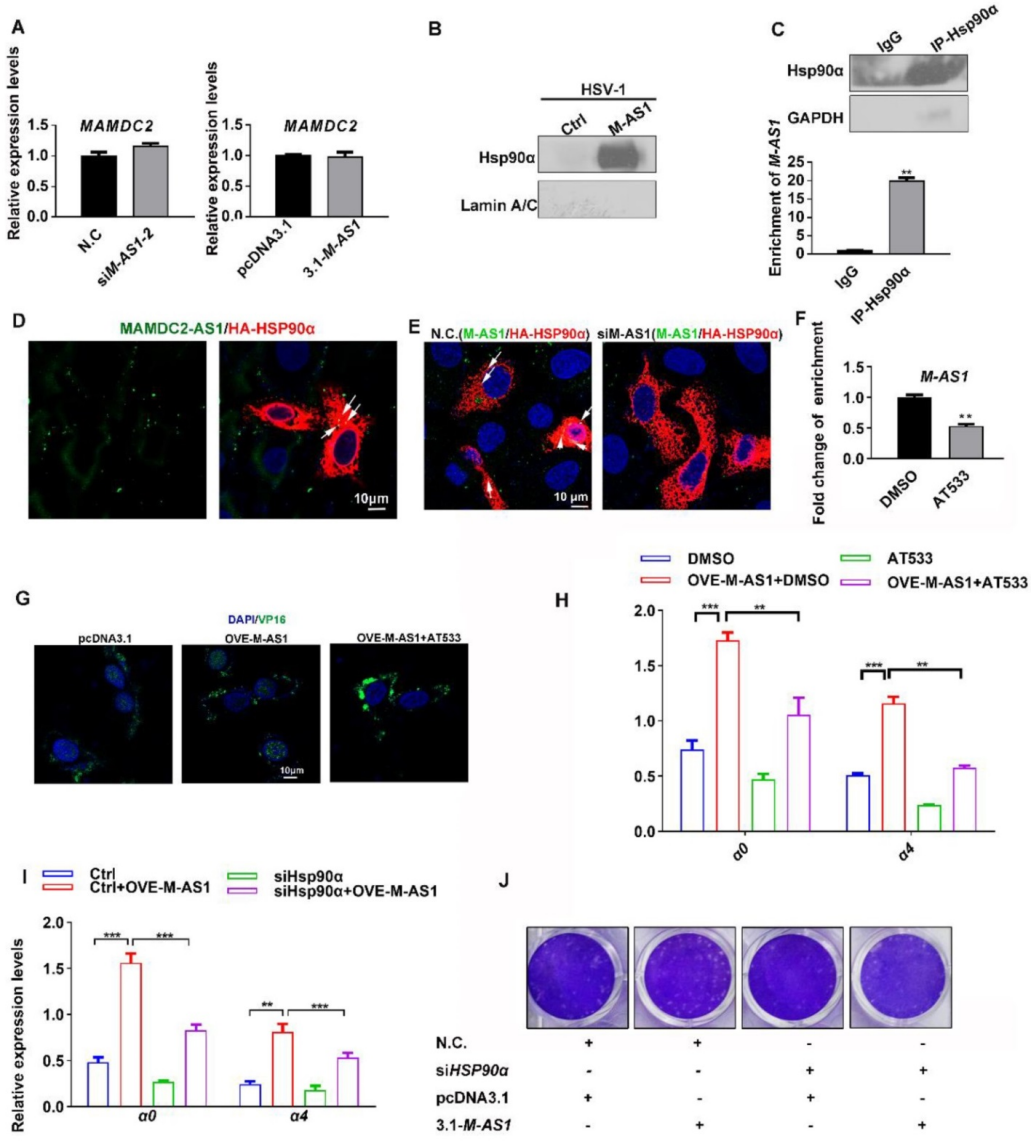
** MAMDC2-AS1 interacts with Hsp90α to facilitate the nuclear import of HSV-1 tegument protein. (A) Left:** HeLa cells were transfected with either indicated MAMDC2-AS1 or N.C. siRNAs (100 nM) and then infected with HSV-1 (MOI 3) for 2 h; **Right**: HeLa cells were transfected with pcDNA3.1-MAMDC2-AS1 or empty vector pcDNA3.1 plasmid (1.5 μg) for 24 h and then infected with HSV-1 (MOI 3) for 2 h. Both samples were collected to extract total RNA for qRT-PCR analysis of the relative RNA levels of MAMDC2. **(B)** RNA pull-down analysis of the binding of MAMDC2-AS1 to Hsp90α and Lamin A/C with the lysates of HeLa cell according to the description in the section of “RNA Pull-Down Assay”. **(C)** Hsp90α RIP followed by qPCR (RIP-qPCR) analysis of co-purified RNA in HeLa cells with HSV-1 infection for 2 h according to the method of “RNA Immunoprecipitation assay”. The efficiency of Hsp90α immunoprecipitation was determined with western blot. **(D)** Confocal microscopy images of MAMDC2-AS1 stained with FISH probe with FITC conjugation (green) combined with immunofluorescence analysis of exogenic Hsp90α (red) expressed from HA in 293T cells. Nucleus were stained with DAPI (blue). **(E)** Confocal microscopy images of MAMDC2-AS1 stained with FITC-conjugated probe (green) combined with immunofluorescence analysis of exogenic Hsp90α (red) expressed from HA in 293T cells with HSV-1 (MOI 3) infection for 2 h. Nucleus were stained with DAPI (blue). **(F)** Hsp90α RIP followed by qPCR (RIP-qPCR) analysis of co-purified RNA in HeLa cells with HSV-1 infection for 2 h in the presence of AT533 (0.5 μM) or DMSO according to the method of “RNA Immunoprecipitation assay”. **(G)** HeLa cells were transfected with pcDNA3.1-MAMDC2-AS1 or empty vector plasmid pcDNA3.1 (1.5 μg) for 24 h and then infected with HSV-1 (MOI 100) at 37°C for 2 h in the presence of AT533 (0.5 μM) or DMSO (control), after which the samples were fixed and VP16 and nuclei labeled with the VP16-specific antibody (green) and DAPI (blue), respectively. Samples were examined and captured using a confocal microscope. **(H)** HeLa cells were transfected with empty vector plasmid pcDNA3.1 (1.5 μg) as a control or pcDNA3.1-MAMDC2-AS1(1.5 μg) for 24 h then infected with HSV-1 (MOI 3) for 2 h in the presence of AT533 (0.5 μM) or DMSO (control), after which total RNA was extracted for detecting the expression levels of the indicated genes by qRT-PCR. **(I)** HeLa cells were co-transfected with siHSP90α (100 nM) and MAMDC2-AS1 plasmid (1.5 μg) for 24 h and then infected with HSV-1 (MOI 3) for 2 h, after which total RNA was extracted for detecting the expression levels of the indicated genes by qRT-PCR. **(J)** HSP90α knockdown reversed the increment of plaque formation units induced by MAMDC2-AS1 overexpression. HeLa cells were co-transfected with N.C. or siHSP90α (100 nM) and pcDNA3.1 or MAMDC2-AS1 plasmid (1.5 μg) as indicated for 24 h and then infected with HSV-1 (MOI 0.01) for 2 h. The cells were then overlaid with medium containing 1% serum, and after 72 h, the plaque formation was examined. All quantitative results were obtained from three independent experiments with three technical replicates per experiments and one typical result was presented as means and S.D.

Two main types of regulation occur between sense and antisense transcripts-concordant regulation and discordant regulation; and most antisense lncRNA function in biological processes by modulating the mRNA expression of the corresponding sense protein-coding genes 38-40. Therefore, we investigated whether MAMDC2-AS1 functions through regulating the expression of *MAMDC2*, a protein-coding gene within the positive-sense strand. However, knockdown of MAMDC2-AS1 did not affect the RNA level of MAMDC2 (**Figure 6A, left**). MAMDC2-AS1 overexpression also did not affect the mRNA expression of *MAMDC2* (**Figure 6A, right**). Collectively, the mechanism of action of MAMDC2- AS1 in HSV-1 infection did not involve MAMDC2. Further given that most lncRNA usually functions through interacting with specific protein, RNA-pull down assay were performed to determine whether MAMDC2-AS1 interacts with the factors that required by the nuclear transport HSV-1, including Hsp90α and Lamin A/C 4, 5, 9, 41. Intriguingly, Hsp90α was identified as an interactor of MAMDC2-AS1*,* but Lamin A/C not (**Figure 6B**). The result of RNA immunoprecipitation (RIP) qRT-PCR further revealed that Hsp90α interacted with MAMDC2-AS1 (**Figure 6C**). Also, the RNA FISH assay indicated that MAMDC2-AS1 colocalized with Hsp90α expressed from plasmids (**Figure 6D**) and this colocalization was disrupted by MAMDC2-AS1 knockdown (**Figure 6E**). Moreover, Hsp90 inhibitor AT533 led to a weakened interaction of MAMDC2-AS1 with Hsp90α as indicated by the result of RIP-qPCR (**Figure 6F**). However, both MAMDC2-AS1 overexpression and knockdown did not change the RNA and protein level of Hsp90α (**Figure S6**). Collectively, the interaction between MAMDC2-AS1 and Hsp90α may facilitate the nuclear import of HSV-1 through enhancing the chaperon function of Hsp90α. We then thus used AT533 to block the chaperon function of Hsp90 then tested the effect of MAMDC2-AS1 on HSV-1 nuclear transport. The confocal image showed that Hsp90 inhibition restored the enhancement of VP16 nuclear import mediated by MAMDC2-AS1 overexpression (**Figure 6G**). Moreover, treatment of Hsp90 inhibitors partly abolished the upregulation of viral IE genes induced by MAMDC2-AS1 overexpression (**Figure 6H**). Knockdown of Hsp90α with siRNA also restored the upregulation of viral IE genes, including *α0* and* α4*, induced by MAMDC2-AS1 overexpression (**Figure 6I**). Consistently, the increment of plaque formation units by MAMDC2-AS1 overexpression can be limited by Hsp90 inhibition (**Figure 6J**)**.** Collectively, the interaction between MAMDC2-AS1 and Hsp90α facilitates the nuclear import of HSV-1 tegument protein VP16.

### YY1 mediates the upregulation of MAMDC2-AS1 upon HSV-1 infection

**Figure 7:**
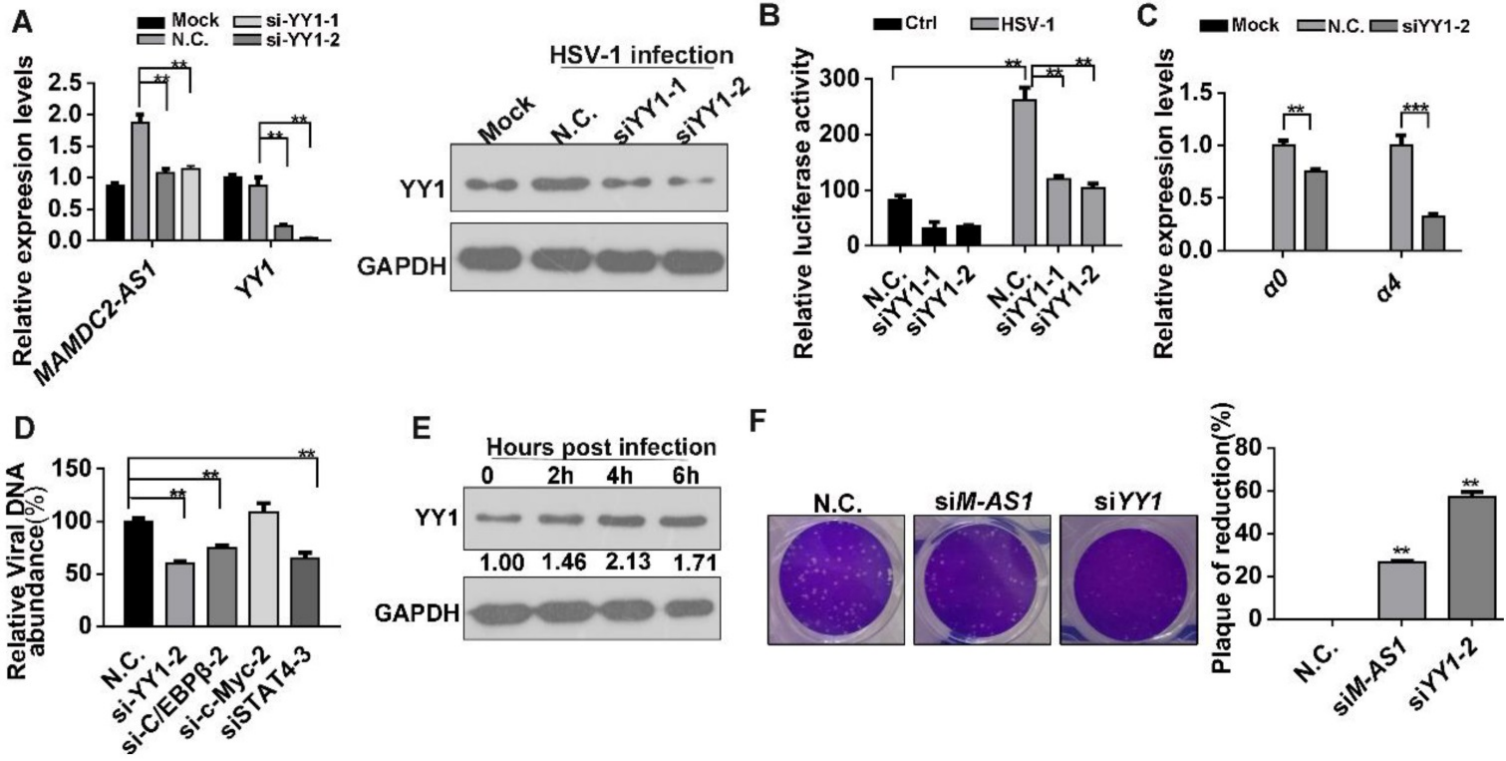
** YY1 mediates the upregulation of MAMDC2-AS1 upon HSV-1 infection. (A)** YY1 knockdown constrained the MAMDC2-AS1 upregulation induced by HSV-1 infection. HeLa cells transfected with N.C. or YY1-specific siRNA (100 nM) for 48 h were infected with HSV-1 (MOI 3) for 3 h. Total RNA was extracted to analyze the level of MAMDC2-AS1 by qRT-PCR. Corresponding protein samples were subjected to immunoblot analysis for the level of YY1; **(B)** YY1 knockdown suppressed the luciferase activity of the MAMDC2-AS1 promoter. HeLa cells were co-transfected with N.C. or YY1-specific siRNA (100 nM), the MAMDC2-AS1 promoter activity reporter plasmids pGL-M-AS1-promoter (500 ng), and the pRL-TK internal-control plasmid (10 ng) for 20 h and then infected with HSV-1 (MOI 3) or not for 3 h. Subsequently, dual-luciferase assays were performed on sample lysates to measure luciferase activity. **(C)** YY1 knockdown in host cells reduced the expression of HSV-1 genes. HeLa cells transfected with N.C. or YY1-specific siRNA (100 nM) for 48 h were infected with HSV-1 (MOI 3) or Mock for another 3 h, after which total RNA was extracted, and qRT-PCR was performed to analyze the expression levels of the HSV-1 genes. **(D)** Knockdown of four predicted transcription factors distinctly affected HSV-1 DNA replication. HeLa cells were transfected with N.C. or siRNAs (100 nM) targeting the indicated transcription factors for 24 h, respectively, and then infected with HSV-1 (MOI 3) for 24 h, after which viral DNA was extracted and then qRT-PCR was performed to measure viral DNA amounts. **(E)** YY1 was significantly upon HSV-1 infection in HeLa cells. HeLa cells were infected with HSV-1 (MOI 3) for the indicated durations, and total-protein extracts were immunoblotted to analyze the levels of YY1. **(F)** YY1 knockdown suppressed the plaque formation induced by HSV-1-infection. HeLa cells were transfected (for 24 h) with siM-AS1-2 (100 nM) or siYY1 (100 nM), after which the cells were infected with HSV-1 (MOI 0.01) for 2 h. The cells were then overlaid with medium containing 1% serum, and after 72 h, the plaque formation was examined (left), and the inhibition ratio of plaque formation were quantified (right). All quantitative results were obtained from three independent experiments with three technical replicates per experiments and one typical result was presented as means and S.D.

To identify the crucial transcription factors (TFs) involving in the induction of MAMDC2-AS1 by HSV-1 infection, several potential TFs were obtained by analyzing the promoter of MAMDC2-AS1 (ranging from 2,000 bp upstream to the start site) through a TFs prediction database (http://alggen.lsi.upc.es/cgi-bin/promo_v3/promo/promoinit.cgi?dirDB=TF_8.3). Next, we selected four TFs that received the highest scores as candidates: C/EBPβ, YY1, STAT4, and c-Myc (**Figure S7**). We transfected siRNAs targeting each of these predicted TFs into HeLa cells, and then detected the induction of MAMDC2-AS1 by HSV-1 infection. The results demonstrated that only YY1 knockdown potently restored the upregulation of MAMDC2-AS1 induced by HSV-1 infection (**Figure 7A and Figure S8**). The efficiency of siRNA against YY1 was determined with qRT-PCR and western-blot (**Figure 7A**). We next cloned the promoter of MAMDC2-AS1 into pGL4.11 [luc2P] to generate a reporter plasmid indicating the activity of *MAMDC2-AS1* promoter. The dual-luciferase assays were then performed and the results indicated that siRNA-mediated knockdown of YY1 suppressed the promoter activity of MAMDC2-AS1 (**Figure 7B**). YY1 knockdown also led to a reduction of the viral IE genes, including* α0* and *α4* (**Figure 7C**), while the knockdown of other predicted TFs did not exert such effects (**Figure S9**). Moreover, YY1 knockdown significantly suppressed the viral genome replication (**Figure 7D**), but knockdown of c/EBP and STAT4 exhibited a minor effect on viral genome replication (**Figure 7D**). Of note, during the process of HSV-1 infection, the protein level of YY1 showed an increment as demonstrated by the results of western blotting (**Figure 7E**). Also, the mRNA level of YY1 showed a times-dependent upregulation upon HSV-1 infection (**Figure S10**). Furthermore, plaque formation assay showed that siRNA-mediated YY1 knockdown limited the plaque formation induced by HSV-1 infection (**Figure 7F**). Based on these results, YY1 is a major TF mediating the upregulation of MAMDC2-AS1 upon HSV-1 infection.

## Discussion

Host cells exhibited a great heterogeneity upon virus infection, while current understanding toward such mechanism remains obscure. The emergence of sing-cell RNA-sequencing provides us to explore the mechanism of host heterogeneity upon virus infection. Prior studies have present the transcriptomics of HDFn during the early stage of HSV-1 infection at single-cell level, highlighting the varying response of host cell upon HSV-1 infection 10, 11. However, the contribution of lncRNA to such heterogeneity remains unknown. Given the importance of lncRNA in virus infection have been gradually recognized, we determine the contribution of lncRNA in the susceptibility of host cells upon HSV-1 infection in this study. Prior HSV-1 sing-cell RNA-sequencing using the reporter virus YFP-HSV-1 that was genetically modified to express ICP4-YFP were re-analyzed and four lncRNAs were found to be overlapped DELs in the comparison of mock group v.s. ICP4-positve group and ICP4-positive v.s. ICP4-negative cells. However, these four lncRNA may not function in the same mechanism as they exhibited an inconsistent effect on HSV-1 IE genes (including α0 and α4) promoter activity and IE genes mRNA expression. In detail, HCG18-001 and CTB-31O20.2-001 did not affect the promoter activity despite their overexpression facilitated the mRNA expression of *α0* and *α4*. In contrast, overexpression of MAMDC2-AS1-201 and NEAT1-002. However, lncRNA NEAT1 was known to facilitate the transcription of HSV-1 IE genes 27, we thus focused on studying MAMDC2-AS1.

Our study demonstrates that HSV-1 infection remarkably stimulates the expression of MAMDC2-AS1 in different human cell lines, including HeLa, HepG2, A549, HFF, and HaCaT, which is consistent with prior RNA-seq results in HSV-1-infected HFFs 16, 19. However, HFF is not selected to perform subsequent study as its basal level of MAMDC2-AS1 is too low, which restricts the RNAi knockdown efficiency. Empirical studies show that lncRNA functions in virus infection mainly through an antiviral response-dependent and antiviral response-independent manner 14, 20, 24, 42. However, the knockdown of MAMDC2-AS1 reduces the expression of multiple antiviral factors upon HSV-1 infection, including *IFITM1*,* IFITM2*,* ISG15*, and *ISG56*. Notably, MAMDC2-AS1 does not affect the level of these factors stimulated by LPS. Therefore, the reduction of antiviral response factors may be a result of the limitation of virus infection but not the cause. Indeed, such mechanism has been reported in a prior influential study revealing a lncRNA suppresses viral infection through regulating host metabolism but not the host innate immune response 43.

We next investigate the direct effect of MAMDC2-AS1 on the HSV-1 life cycle. After excluding the possibility of MAMDC2-AS1 affecting viral attachment or cell internalization, we determine the effect of MAMDC2-AS1 on the subsequent phases of HSV-1 life cycle, including nuclear transport of viral tegument proteins. Both of Hsp90α and lamina are already known factors participating in the nuclear transport of HSV-1 5, 6, 9, 41. We find that MAMDC2-AS1 interacts with Hsp90α, but not the nuclear lamina, to enhance its chaperone function to facilitate the nuclear import of HSV-1 tegument protein VP16. Treatment of Hsp90 inhibitors AT533 largely restored the function of MAMDC2-AS1 on the nuclear transport and IE genes expression of HSV-1, as well as the increment of plaque formation units generated by HSV-1 infection. The function of Hsp90α-interacting lncRNA in virus infection are reported here for the first time, despite the interacting sites between MAMDC2-AS1 and Hsp90α need to be further identified. In this study, we found that MAMDC2-AS1 does not affect the mRNA expression of MAMDC2, a corresponding coding gene within positive strand. The mechanism is distinct from the common regulated mechanism of antisense transcript that usually affect the level of corresponding protein-coding mRNA, such as β-secretase-1 (BACE1)-antisense transcript (BACE1-AS1) and KRT7-AS1 38, 40.

We also identify YY1 as a main transcription factor mediating the upregulation of MAMDC2-AS1 upon HSV-1 infection; however, we cannot currently exclude the possibility that other transcription factors also participate in this process, given that we only select four potential transcription factors for our analyses. Intriguingly, YY1 knockdown more potently suppress plaque formation induced by HSV-1 infection than do MAMDC2-AS1 knockdown alone. Such effect can be explained by several reasons. For one thing, YY1 is known to bind to the promoter and internal sites of leaky-late kinetic genes of HSV-1 to enable their maximal transactivation 44, 45. For another, a transcription factor is always involved in the regulation of multiple genes, which suggests that YY1 may modulate other genes that participated in the modulation of HSV-1 infection. Moreover, protein inhibition of activated STAT4 is reported to restrict HSV-1 infection through the cellular intrinsic antiviral immune response 46, and in this regard, we observe that STAT4 knockdown, followed by HSV-1 infection, limits viral gene expression and genome replication. However, further investigation is required to elucidate the function of MAMDC2-AS1 *in vivo*, because several lncRNAs, such as NEAT1, play distinct roles in the antiviral response and virus life cycle, which could produce divergent effects *in vitro* and *in vivo* 27-29.

## Materials and Methods

### Cells and viruses

HaCaT cells (CRL-2310), HepG2 cells (HB-8065), HFF cells, and HeLa cells (CCL-2) were obtained from the American Type Culture Collection (ATCC; Manassas, VA, USA) and cultured in DMEM supplemented with 10% fetal calf serum. Human 293T cells were cultured according to the description in our prior study 5, 47. All cells were incubated at 37°C in a humidified atmosphere with 5% CO_2_, and the medium was replaced every 2 days. A549 cells (ATCC, CCL-185) were cultured in RPMI 1640 medium supplemented with 10% fetal calf serum. All cells were grown and maintained at 37°C and 5% CO_2_ in a humidified incubator. HSV-1 strain F was obtained from Hong Kong University, further propagated in Vero cells, and then stored at -80°C until use. The reporter virus EGFP-HSV-1, which expresses EGFP fused with U_S_11, was a gift from Prof. Kurihara Hiroshi (College of Pharmacy, Jinan University) and was cultured under the same conditions with our prior study 5.

### Data analysis of single-cell RNA-sequencing

The Frasergen company (Wuhan, China) provides us essential help for analyzing the differentially expressed lncRNA genes towards a deposited single-cell RNA-sequencing by prior study 10. Briefly, the raw data of single-cell RNA- sequencing (accession number GSE126042) in fastq format were obtained from Gene Expression Omnibus (GEO). The low-quality reads were filtered by FastQC software with default argument to obtain clean data. All the downstream analyses were based on the clean data with high quality. Mapping the filtered RNA-seq data to human genome (Homo sapiens. GRCh38) using HISAT2 v2.0.4 48. HISAT2 was run with '--rna-strandness RF', other parameters were set as default. The mapped reads of each sample were assembled by StringTie (v1.3.1) in a reference-based approach 49. StringTie uses a novel network flow algorithm as well as an optional de novo assembly step to assemble and quantitate full length transcripts representing multiple splice variants for each gene locus. We used CNCI software and Coding Potential Calculator to predict the coding capacity of candidate lncRNAs 50, 51. The differentially expressed lncRNA were counted with Cuffdiff and the transcripts with a P-adjust <0.05 and fold-change ≥2 were assigned as differentially expressed 52. Cuffdiff provides statistical routines for determining differential expression in digital transcript or gene expression data using a model based on the negative binomial distribution 52.

### Inhibitors, antibodies, siRNAs, and plasmids

All antibodies used in this study were from commercial sources: anti-HSV1/HSV2 ICP5 Major Capsid Protein (ab6508), anti-Hsp90α (ab59459), anti- HSV1/2 VP16 (ab11026) and anti-histone H3 (ab1791) (Abcam, Cambridge, UK); anti-GAPDH (2118), anti-LC3B (2775), and anti-HA (3956) (Cell Signaling Technology, MA, USA); anti-Lamin A/C(10298-1-AP) (Proteintech, Wuhan, Hubei, China); anti-Oct-1 (sc-8024) and anti-YY1 (sc-7341) (Santa Cruz Biotechnology, CA, USA); and Alexa Fluor 488/594-conjugated goat anti-mouse or anti-rabbit IgG (Invitrogen, Carlsbad, CA, USA).

All the lncRNA expressing plasmids were generated by Tsingke company (Beijing, China) unless otherwise specifically mentioned. Shortly, the full length of lncRNA was synthesized by Tsingke company then cloned into the empty vector pcDNA3.1(+) plasmids. The plasmids pcDNA3.1(+)*-MAMDC2-AS1* were purchased from Vigene Biosciences (Jinan, Shandong, China). To construct U_L_25 expressing plasmid pCMV-HA-U_L_25, the cDNA obtained from HSV-1-infected HeLa cells was used as a template to amplify the coding sequence of U_L_25 then cloned into pCMV-HA vector. All constructed plasmids were verified based on DNA sequences obtained by Sanger sequencing in Tsingke company. All the primer of constructing plasmids in this study can be obtained from **Table S1**.

Latrunculin A (Lat-A), sequesters actin monomers, was purchased from Invitrogen as demonstrated in our prior study 33. The source of Hsp90 inhibitor AT533 also has been described in our prior study 5. All siRNAs were purchased from Gene Pharma (Shanghai, China); siRNA sequence details are provided in **Table S2**. The cytotoxicity of drugs and siRNAs and transient expression were detected using 3-(4,5-dimethyl-2-thiazolyl)-2,5-diphenyl-2H-tetrazolium bromide assays; all inhibitors and siRNA were used at noncytotoxic concentrations.

### RNA FISH and immunofluorescence microscopy

The RNA FISH assay was performed according to the user manual instructions (Ribobio, Guangzhou, China). Briefly, a MAMDC2-AS1-specific probe was designed and synthesized by Ribobio company. The probe was conjugated with Cy3 or FITC. Cells were infected with HSV-1 for indicated times, washed thrice with PBS, fixed in 4% paraformaldehyde for 10 min, washed thrice with PBS, permeabilized with precooled 0.5% Triton X-100 (Sigma-Aldrich) for 5 min, and then washed thrice again with PBS. After cells were blocked with the pre-hybridization buffer at 37°C for 30 min, they were labeled with the MAMDC2-AS1 FISH probe (mixed in dilution buffer) overnight at 37°C in the dark. All subsequent steps were performed in the dark. The cells were washed thrice with buffer I (4× SSC, 0.1% Tween-20) to reduce the background signal, and then sequentially washed (once each) with solution buffers II and III. After a 5 min wash with PBS, 1 mg/ml 4',6-diamidino-2- phenylindole (DAPI; Biotium, Fremont, CA, USA) was added for 15 min to label nuclei, and cells were then washed and mounted for observation. For observing the colocalization of MAMDC2-AS1 with Hsp90α, the antibody against Hsp90α were incubated at room temperature for 1 hour before labeling nuclei. Fluorescence images were captured using a Zeiss LSM510 Meta confocal system equipped with a 63× oil-immersion objective lens (Carl Zeiss, Oberkochen, Germany). The fluorescence intensity of acquired images was analyzed and quantified using Zen software (Carl Zeiss).

For detecting ICP5 or VP16 localization, our previously described protocol was performed with minor modifications 33. Briefly, to determine the effect on virus attachment by MAMDC2-AS1, cells were challenged with HSV-1 at 4°C for 1 h and then treated with proteinase K (2 mg/ml, Beyotime, Suzhou, China) for another 1 h at 4°C to remove extracellular viral particles. Further proteolysis was inhibited by adding phenylmethylsulfonyl fluoride (PMSF; 1 mM, Beyotime) and 3% (w/v) BSA in PBS. To test the effect on virus internalization by MAMDC2-AS1, cells were challenged with HSV-1 at 4°C for 1 h and then incubated at 37°C for indicated durations. Samples after the indicated treatments were washed, fixed, permeabilized, and blocked. The cells were then incubated with the anti-ICP5 (1:200) or anti-VP16 (1:100) antibody overnight at 4°C, followed by incubation with the Alexa Fluor 488-conjugated secondary antibody (1:1000) at room temperature for 1 h. The cells were also stained with 5 μM tetramethyl rhodamine isocyanate-phalloidin (Sigma; 40 min) and 1 mg/ml DAPI (10 min) to label F-actin and nuclei, respectively. Subsequently, the samples were examined as described in the case of the FISH assays. Final images were captured by the Zeiss LSM510 Meta confocal system with identical detection volumes for different color channels for different samples.

### Transfection

All cells were plated the day before transfection at a density of 2.0×10^5^/mL, and were at 60-80% confluence at the time of transfection. All transfections were performed using the jetPRIME transfection reagent (Polypus Transfection, Strasbourg, France) according to the manufacturer's instructions. Briefly, the indicated amounts of plasmids or siRNAs were diluted with jetPRIME buffer in a corresponding volume (as per the instructions), gently vortexed for 10 s, mixed with the transfection reagent, vortexed again for 10 s to mix thoroughly, and then spun down and incubated for 10 min at room temperature. Finally, the transfection mixture was gently added to cells cultured in serum-containing medium. To maintain cell viability, the medium containing the transfection reagent was replaced with growth medium after transfection for 6 h.

### Viral plaque assay

Virus titers were determined based on cytopathic effects in Vero cells (as previously reported) to calculate the 50% tissue culture infectious dose (TCID_50_) 5. Reduction of plaque formation was measured to determine the appropriate dilution, which was then used in the plaque assays, as previously described 53. Specifically, Vero cells were seeded in 24-well plates at a density of 4 × 10^5^/well. Next, cells were adsorbed with HSV-1 (at various dilutions) for 2 h. The virus inoculum was removed, and overlay medium (maintenance medium containing 1% methylcellulose) was added to each well. After 72 h of incubation, cell monolayers were fixed with 10% formalin and stained with 1% crystal violet. Plaques were counted with the plaque number at the appropriate dilution of approximately 30-50 plaques/well. For testing the effect of MAMDC2-AS1 on plaque formation units, cells were seeded in 24-well plates at a density of approximately 2×10^5^/well and then transfected with siRNAs or plasmids for 24 h. Next, the cells were adsorbed with HSV-1 at previously confirmed dilutions for 2 h, after which the virus inoculum was removed and overlay medium (maintenance medium containing 1% methylcellulose) added to each well. After 72 h of incubation, cell monolayers were fixed with 10% formalin and stained with 1% crystal violet. Plaques were counted, and the average number of plaques was calculated.

### Detection of HSV-1 binding and internalization

The amount of virus binding to and internalized by cells was measured as described previously 32-34, with minor modifications. HSV-1 at a low genome/PFU ratio, obtained by treating with DNase I (Beyotime) for 30 min at 37°C, was used to ensure a high correlation between the number of viral genomes and the concentration of viral particles 54.

To determine the amount of virus binding, cells were seeded 24 h prior to the experiment in 12-well plates at a density of 2 × 10^5^/well and then inoculated with HSV-1 for 1 h at 4°C. HSV-1 binding to cells was quantified after removing extracellular viral particles by treatment with proteinase K (2 mg/ml, Beyotime) for 1 h at 4°C. Proteinase K was added to cells after virus binding at 4°C to measure the surface-bound virions that could not be removed by the proteinase K treatment, and further proteolysis was inhibited by adding PMSF (1 mM; Beyotime) and 3% (w/v) BSA in PBS. The cells were pelleted at 1,500 rpm for 15 min, and the DNA was extracted from the samples by using the TIANamp Virus DNA/RNA kit (Tiangen, Beijing, China) to determine viral DNA copy numbers using qRT-PCR-based detection of the viral *U_L_47* gene.

To measure the amount of viral entry, cells were seeded 24 h prior to the experiment in 12-well plates at a density of 4 × 10^5^/well and then inoculated with HSV-1 for 30 min at 37°C. The HSV-1 internalized by cells was quantified after extracellular viral particles and virions that bound to but did not enter the cells were removed by washing with PBS (pH 3.0). The cells were collected, and viral DNA was extracted, after which the same protocols as those described above were performed.

For absolute quantitative analysis, a serial dilution of the plasmid pcDNA3.1-VP16 (*U_L_48*) constructed in our prior study 5 was used as a standard. The initial copy number of the *U_L_48* gene in each group was calculated using the following formula: C_T_ = -K logX_0_ + b, where C_T_ is the cycle threshold, and K, X_0_, and b are the slope, initial copy number, and constant, respectively.

### Western blotting

Cell samples were rinsed thrice with precooled PBS and lysed in SDS buffer (Beyotime) containing 1 mM PMSF (Beyotime). Lysates were heated at 100°C for 15 min. Insoluble cell debris was discarded following centrifugation at 12,000 × *g* for 10 min at 4°C. The protein concentrations of retained supernatant was measured using an enhanced bicinchoninic acid (BCA) protein assay kit (Beyotime). Subsequently, the lysates were mixed with calculated volumes of 5× SDS-PAGE buffer (Beyotime) and SDS buffer to obtain equivalent protein concentrations and then boiled for 10 min. Finally, samples were separated using 8-10% gradient SDS-PAGE, transferred to polyvinylidene fluoride membranes (Merck Millipore, Darmstadt, Germany), and probed with indicated primary antibodies and then with HRP-conjugated secondary antibodies (Invitrogen). Immunoreactive bands were detected using enhanced chemiluminescence (Millipore), and the band intensity of each target protein was calculated using Quantify One software (Bio-Rad, Hercules, CA, USA) and normalized to GAPDH band intensity.

### RNA Pull-Down Assay

RNA pull-down assay was performed according to the user guidelines of pierce magnetic RNA-Protein Pull-Down Kit (Thermal Fish, 20164) with minor revision. Briefly and firstly, purified MAMDC2-AS1 were *in vitro* transcribed by Takara (Takara, Dalian, China). Next, the Pierce RNA 3' Desthiobiotinylation Kit (Thermal Fish, 20163) were used to generate the biotin-labelled MAMDC2-AS1. To ensure the formation of RNA second structure, the newly synthesized RNA was heated to 95°C for 2 min and put an ice for another 2 min, then were left at room temperature for 20 min. The streptavidin magnetic beads were incubated with biotin-labeled MAMDC2- AS1 to obtain the protein factors interacting MAMDC2-AS1. The fold beads-biotin labelled MAMDC2-AS1 was then incubated with HeLa lysis (containing 2 mg proteins) in RIP buffer at 4°C with agitation or rotation for 2 hours to achieve RNA-binding proteins. Beads were washed six times with RIP wash buffer and final retrieved proteins were boiled in SDS loading buffer, followed by the analysis of Western blotting.

### RNA Immunoprecipitation assay

The standard RNA immunoprecipitation (RIP) protocol were according to the manual of magna RIP Kit (17-700, Millipore). In brief, indicated cell lysis in buffer containing RNase inhibitor was centrifugated minorly to remove cell debris and the supernatants were obtained then remove 10 µL of the supernatant into a new tube and label as 10% Input. Remaining supernatants were immunoprecipitated with the magnetic beads conjugating Hsp90α antibody or IgG overnight at 4°C. The immunoprecipitation production was washed five times then digested with proteinase K containing buffer. The immunoprecipitated RNA and input RNA samples was purified using phenol: chloroform: isoamyl alcohol (125:24:1 pH=4.3) (Aladdin, P120619) and ethanol precipitation, and was subjected to analyze the abundance of MAMDC2-AS1 with one step qRT-PCR assay (Takara).

### RNA extraction and real-time quantitative PCR

Cells were infected with HSV-1 for various durations, and total RNA was extracted using TRIzol reagent (Tiangen) according to the manufacturer's instructions. RNA concentration was measured using a NanoPhotometer P330 (IMPLEN, Munich, Germany) at 260 nm/280 nm, and 1 μg of RNA was then reverse-transcribed into cDNA using a PrimeScript RT reagent kit (Takara). Of note, the lncRcute lncRNA First-Strand cDNA Synthesis kit (with gDNase) (Tiangen) was used to generate the first stand of the lncRNA, given that the basal level of MAMDC2-AS1 was low. The reverse-transcription products were diluted fivefold and analyzed using qRT-PCR; the real-time assay was performed using a Bio-Rad CFX96 real-time PCR system (Bio-Rad), and each primer (Sango, Shanghai, China) was used at 250 nM 8. The levels of mRNA transcripts were standardized against those of the housekeeping gene *GAPDH* with the 2^-ΔΔCT^ method using the CFX96 system software (Bio-Rad). Gene-specific primer pairs used in the qPCR assay are described in **Table S3**.

### RNA subcellular isolation

Cells were harvested and washed with ice-cold PBS twice. After centrifugation 1000 g for 5 min, supernatants were removed. Cell pellets were resuspended with 100 μL 0.1% v/v NP40 in RNase free water containing 10 mM Ribonucleoside Vanadyl Complex by pipetting gently. After centrifugation with 1000 × g for 5min, the supernatant was collected and labeled as the cytoplasmic fraction and the pellet was washed with 200 μL ice-cold 0.1% NP40-PBS for three times. The supernatant was discarded and the pellet was labeled as nucleus. Next, 1 mL TRIzol was added to both nucleus and cytoplasm fraction and RNA was extracted according to the manufacturer's protocol.

### Identification of VP16 levels in the cytoplasm and nucleus

To separate cytoplasmic and nuclear components, cells were lysed using a nuclear and cytoplasmic protein extraction kit (Beyotime) according to the manufacturer's instructions. Specifically, indicated samples were lysed with cytoplasmic lysis buffer containing 1 mM PMSF and centrifuged at 12,000 × *g* for 5 min at 4°C. The clarified supernatants were collected as the cytoplasmic fraction. Next, the precipitates were further lysed with the nuclear lysis buffer containing 1 mM PMSF at 4°C for 30 min and vortexed once every 2 min during lysis on ice. This was followed by centrifugation at 12,000 × *g* for 5 min at 4°C, and the clarified supernatants were collected as the nuclear fraction. Protein concentrations were determined using the enhanced BCA protein assay kit, and then, the cytoplasmic and nuclear components were analyzed by western blotting. Levels of GAPDH and histone protein H3, as markers for the cytoplasm and nucleus, respectively, were assessed using specific monoclonal antibodies.

### Dual-luciferase reporter assays

Dual-luciferase assays were performed using a Dual Luciferase Reporter Assay System (E1910; Promega, Madison, WI, USA) according to our prior published studies 5. Briefly, cells were transfected with *MAMDC2-AS1*-specific siRNAs and the reporter plasmids containing the target promoters (pGL4.12 [luc2p]-*α0* promoter (pGL-*α0*) and pGL4.12 [luc2p]-*α4* promoter (pGL-*α4*)), together with the plasmid pRL-TK expressing *Renilla* luciferase as an internal control to normalize for transfection efficiency. At 24 h post transfection, firefly and *Renilla* luciferase activities were measured at Jinan University according to the instructions provided with assay kit. Relative luciferase activity (RLA) was determined by normalizing to the *Renilla* luciferase activity. Similarly, the promoter activity of *MAMDC2-AS1* was also assessed using this system in relation to the extent of siRNA-mediated knockdown of indicated transcription factors. Each experiment was repeated thrice, and the mean was calculated for statistical analysis.

### Statistical analysis

All data of qPCR present in this manuscript were representative or statistics (mean ± SD) of the results from at least three independent experiments. Student's two-tailed t test was used for all statistical analysis and performed by GraphPad Prism 8 software, with the level of significance set at (∗∗) p < 0.01, and (∗) p < 0.05.

## Supplementary Material

Supplementary figures and tables.
