# Supplementary material for: Single-cell RNA-sequencing analysis identifies host long noncoding RNA MAMDC2-AS1 as a co-factor for HSV-1 nuclear transport
Source: Int J Biol Sci. 2020 Mar 5;16(9):1586–603. doi: 10.7150/ijbs.42556 (PMC7097924; doi:10.7150/ijbs.42556)
Supplement: Supplementary file 1 — Supplementary figures and tables. [file ijbsv16p1586s1.pdf]

Supplementary tables.

Table S1. List of primers used in constructing plasmids.

Plasmids	Restriction enzyme sites	Primers (forward)	Primers (reverse)
pCMV-HA-UL25	EcoRI and XhoI	CGGAATTCAAATGGACCCGTACTGCCCATTTGACG	CCGCTCGAGCTAAACCGCCGACAGGT

Table S2. Sequence of siRNAs used in our study.

Name	Targeting Sequence
si-YY1-1	Sense: GCCUCUCCUUUGUAUAUUAUU
	Antisense: AAUAAUAUACAAAGGAGAGGC
si-YY1-2	Sense: CCCAAACAACUGGCAGAAUUU
	Antisense: AAAUUCUGCCAGUUGUUUGGG
si-YY1-3	Sense: CCUCCUGAUUAUUCAGAAUAU
	Antisense: AUAUUCUGAAUAAUCAGGAGG
si-STAT4-1	Sense: CGCACCAAGAAAGGAAGCAAA
	Antisense: UUUGCUUCCUUUCUUGGUGCG
si-STAT4-2	Sense: GCGAGACUACAAAGUUAUUAU
	Antisense: AUAAUAACUUUGUAGUCUCGC
si-STAT4-3	Sense: GCUUUACACUAUUGGCAGAAA
	Antisense: UUUCUGCCAAUAGUGUAAAGC
si-C/EBPβ-1	Sense: UGGUGUUAUUUAAAGAA
	Antisense: UUCUUUAAAUAACACCA
si-C/EBPβ-2	Sense: ACAAGCACAGCGACGAGUACA
	Antisense: UGUACUCGUCGCUGUGCUUGU
si-C/EBPβ-3	Sense: CACCCUGCGGAACUUGUUCAA
	Antisense: UUGAACAAGUUCCGCAGGGUG
si-c-Myc-1	Sense: CCUGAGACAGAUCAGCAACAA

	Antisense: UUGUUGCUGAUCUGUCUCAGG
si-c-Myc-2	Sense: CAGUUGAAACACAAACUUGAA
	Antisense: UUCAAGUUUGUGUUUCAACUG
siHsp90α-2	Sense: AAUAUCGUCGGGAUUUCUGGU
	Antisense: CAGAAAUCCCGACGAUAUUA
si-MAMDC2-AS1-235	Sense: GCAUUCCUCGUUUGAAUAA
	Antisense: UUAUUCAAACGAGGAAUGC
si-MAMDC2-AS1-2	Sense: GCGCGCGUACGAAAAACAAUUACGG
	Antisense: CCGUAAUUGUUUUUCGUACGCGCGC
Negative Control (N.C.)	Sense: UUCUCCGAACGUGUCACGUTT
	Antisense: ACGUGACACGUUCGGAGAATT

Table S3. List of primers used in our qRT-PCR experiment.

Gene	Primers (forward)	Primers (reverse)
α0	CCCACTATCAGGTACACCAGCTT	CTGCGCTGCGACACCTT
α4	CGACACGGATCCACGACCC	GATCCCCCTCCCGCGCTTCGTCCG
$U_{L}23$	ACGATGATGATGAGGTTCCC	CAGCTCCTCTAGGAACAGCG
$U_{L}29$	ATGAACAGCTGCAACGGGTA	GTCGTTACCGAGGGCTTCAA
HSP90α	AGTCTGGGACCAAAGCGTTC	ACTGTGAATGATCCCCCTGC
LMNA	CTTCTGCCTCCAGTGTCACG	CCCATCTCTTGTATGATGCTGC
GAPDH	CACCATCTTCCAGGAGCGAG	AGAGGGGGCAGAGATGATGA
U6	CTCGCTTCGGCAGCACA	AACGCTTCACGAATTTGCGT
$U_{L}47$	ACGATGATGATGAGGTTCCC	CAGCTCCTCTAGGAACAGCG
MAMDC2	CACTACTGGGGTAGGCTACTA	AGCCAGGAAATGCTCTGTTCA
M2-AS1	TTGCTTATAGCCCACCCACG	TTTGTGGCCCTCCCATTTCA
IFITM1	ACTAGTAGCCGCCCATAGCC	GCACGTGCACTTTATTGAATG
IFITM2	ATGTGGTCTGGTCCCTGTTC	CATGAAGATGCCCAAAATCA
ISG15	AGTGTCCCAGAGTTCATTTTTG	GTCGCCCAGGCTGATCTC

ISG56	ACCACAGAGAAAAAGCAGGACC	ACCATTTGTACACATCTCCACTGT
TNFα	CCCCAGGGACCTCTCTCTAA	CAGCTTGAGGGTTTGCTACA
YY1	AAGCCCTTTCAGTGCACGTT	TCTCCGGTATGGATTCGCAC
c-Myc	TCCCTCCACTCGGAAGGACTA	GCTGGTGCATTTTCGGTTGT
c/EBPβ	TTTGTCCAAACCAACCGCAC	GCATCAACTTCGAAACCGGC
STAT4	AGTAGGAGGAGGCTAGGTCAG	GGATGGGTAGCCAGGATCAAA

Supplementary figure legends.

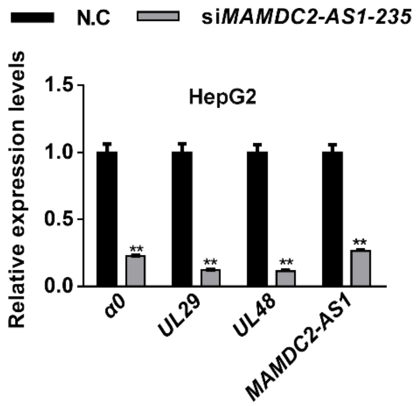

Figure S1. MAMDC2-AS1 knockdown reduces the expression of HSV-1 genes in HepG2 cells. HepG2 cells transfected with MAMDC2-AS1-targeting siRNA (siMAMDC2-AS1) or with a N.C. siRNA (100 nM), and then infected with HSV-1 (MOI 3) for 10 h. Total RNA was extracted to analyze the level of indicated genes by qRT-PCR.

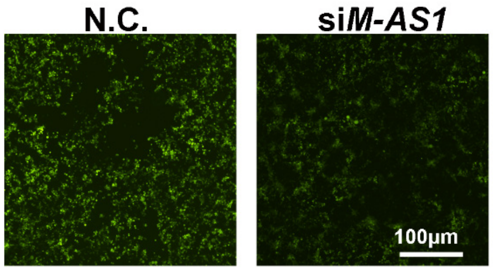

Figure S2. MAMDC2-AS1 knockdown reduces the fluorescence intensity of EGFP-HSV-1 infected HeLa cells. HeLa cells transfected with MAMDC2-AS1-targeting or with a N.C. siRNA (100 nM) for 24 h were infected with EGFP-HSV-1 (MOI 1) for another 24 h. The cells were observed with fluorescence microscope.

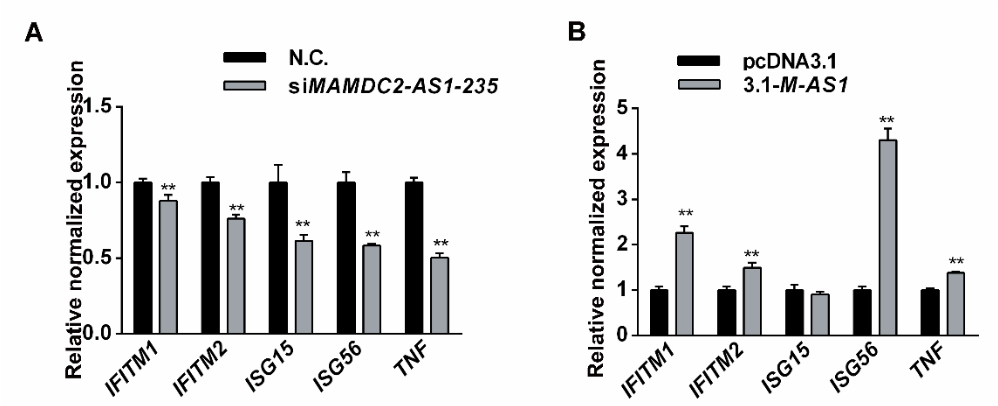

Figure S3. (A) MAMDC2-AS1 knockdown reduced the expression of antiviral response factors. HeLa cells were transfected with MAMDC2-AS1 siRNA or with a N.C. siRNA (100 nM) for 24 h and then infected with HSV-1 (MOI 3) for 4h; subsequently, total RNA was extracted for analyzing the level of the indicated genes by qRT-PCR. (B) Increased expression of antiviral response genes in the context of MAMDC2-AS1 overexpression. HeLa cells were transfected with pcDNA3.1-MAMDC2-AS1 or empty vector pcDNA3.1 plasmid (1.5 μg) for 24 h and infected with HSV-1 (MOI 3) for 4 h, after which total RNA was extracted and subjected to qRT-PCR analysis for determining the relative level of the indicated genes.

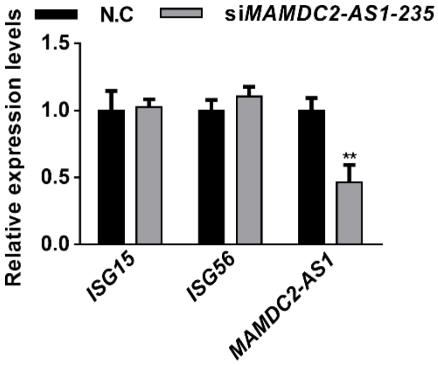

Figure S4. MAMDC2-AS1 knockdown is failed to affect the level of interferon stimulated genes upon LPS stimulation. HeLa cells were transfected with MAMDC2-AS1 siRNA (100 nM) or with a N.C. siRNA (100 nM) for 24 h and then stimulated with LPS (100 ng/mL) for 3 h. Subsequently, total RNA was extracted for analyzing the level of the indicated genes by qRT-PCR.

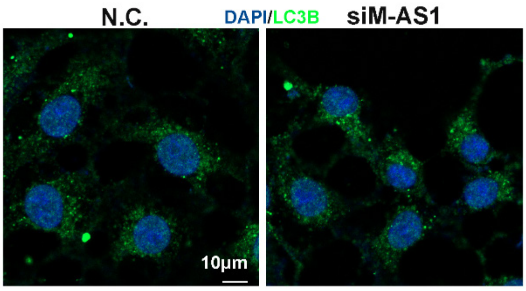

Figure S5. MAMDC2-AS1 knockdown is failed to affect autophagy. HeLa cells were transfected with MAMDC2-AS1-targeting siRNA (100 nM) or with a N.C. siRNA (100 nM) for 24 h and then infected with HSV-1 (MOI 3) for 4h, after which the cells were fixed and LC3B and nuclei were labeled with LC3B-specific antibody (green) and DAPI (blue), respectively.

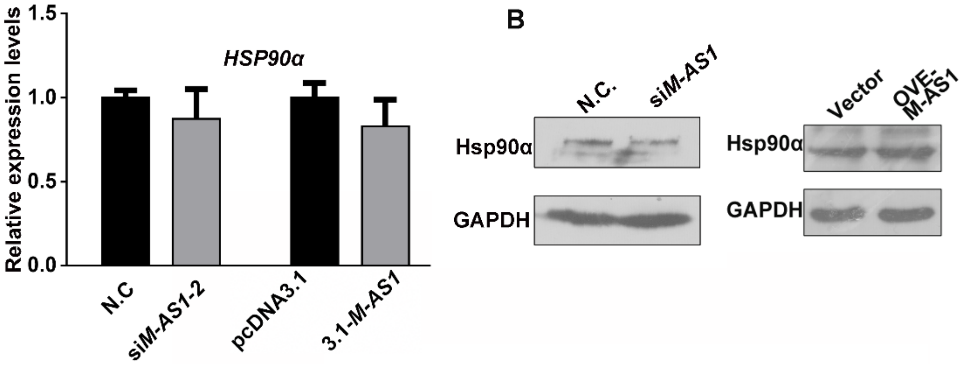

Figure S6. (A) The samples from Figure 6.A were collected to extract total RNA for qRT-PCR analysis of the relative RNA levels of Hsp90α. (B) The samples from Figure 6A were collected to obtain total-protein then subjected to analyze with western blotting to detect the levels of Hsp90α.

0	C/EBPbeta [T00581]	1	YY1 [T00915]	2	GR -beta [T01920]	3	GR -alpha [T00337]	4	c-Myc [T00140]	5	GATA -1 [T00306]	6	GR [T05076]	7	TFII -I [T00824]
8	c-Jun [T00133]	9	IRF -2 [T01491]	10	NF -Y [T00150]	11	STAT4 [T01577]	12	TFIID [T00820]	13	AP -2alphaA [T00035]	14	Pax -5 [T00070]	15	HNF -3alpha [T02512]
16	FOXP3 [T04280]	17	XBP -1 [T00902]	18	PXR -1: RXR -alpha [T05671]	19	ER -alpha [T00261]	20	RXR -alpha [T01345]	21	SRY [T00997]	22	PR B [T00696]	23	PR A [T01661]
24	NF -1 [T00539]	25	TCF -4E [T02878]												

Figure S7. Potential transcription factors by using a prediction database

(http://alggen.lsi.upc.es/cgi-bin/promo_v3/promo/promoinit.cgi?dirDB=TF_8.3) for analyzing the promoter sequences of MAMDC2-AS1 (ranging from 2,000 bp upstream to the start site), which were obtained from the UCSC database.

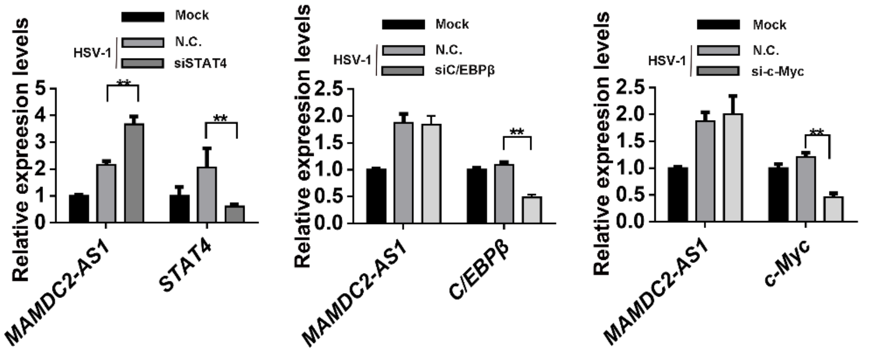

Figure S8. HeLa cells were transfected with siRNAs (100 nM) or with a N.C. siRNA (100 nM) targeting the indicated transcription factors for 48 h and then infected with HSV-1 (MOI 3) for 3 h, after which qRT-PCR was performed to measure the level of MAMDC2-AS1.

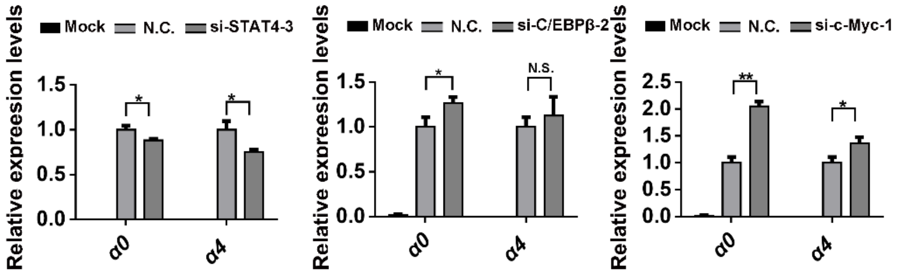

Figure S9. HeLa cells were transfected with siRNAs (100 nM) or with a N.C. siRNA (100 nM) targeting the indicated transcription factors for 48 h and then infected with HSV-1 (MOI 3) for 3 h, after which qRT-PCR was performed to measure the expression of viral indicated genes.

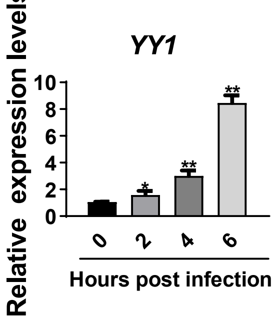

Figure S10. HeLa cells were infected with HSV-1 (MOI 3) for the indicated durations, and total-RNA were extracted to analyze the mRNA expression of YY1 with qRT-PCR.
